# Retuning of Lexical-Semantic Representations: Repetition and Spacing Effects in Word-Meaning Priming

**DOI:** 10.1037/xlm0000507

**Published:** 2017-12-28

**Authors:** Hannah N. Betts, Rebecca A. Gilbert, Zhenguang G. Cai, Zainab B. Okedara, Jennifer M. Rodd

**Affiliations:** 1 Department of Experimental Psychology, University College London; 2 Department of Experimental Psychology, University College London, and School of Psychology, University of East Anglia; 3 Department of Experimental Psychology, University College London

**Keywords:** lexical ambiguity, lexical-semantic representations, priming, repetitions, spacing

## Abstract

Current models of word-meaning access typically assume that lexical-semantic representations of ambiguous words (e.g., ‘*bark* of the dog/tree’) reach a relatively stable state in adulthood, with only the relative frequencies of meanings and immediate sentence context determining meaning preference. However, recent experience also affects interpretation: recently encountered word-meanings become more readily available (Rodd et al., 2016, 2013). Here, 3 experiments investigated how multiple encounters with word-meanings influence the subsequent interpretation of these ambiguous words. Participants heard ambiguous words contextually-disambiguated towards a particular meaning and, after a 20- to 30-min delay, interpretations of the words were tested in isolation. We replicate the finding that 1 encounter with an ambiguous word biased the later interpretation of this word towards the primed meaning for both subordinate (Experiments 1, 2, 3) and dominant meanings (Experiment 1). In addition, for the first time, we show cumulative effects of multiple repetitions of both the same and different meanings. The effect of a single subordinate exposure persisted after a subsequent encounter with the dominant meaning, compared to a dominant exposure alone (Experiment 1). Furthermore, 3 subordinate word-meaning repetitions provided an additional boost to priming compared to 1, although only when their presentation was spaced (Experiments 2, 3); massed repetitions provided no such boost (Experiments 1, 3). These findings indicate that comprehension is guided by the collective effect of multiple recently activated meanings and that the spacing of these activations is key to producing lasting updates to the lexical-semantic network.

Efficient language communication involves keeping track of the meanings of words that have been used or encountered recently (e.g., encountering ‘bark’ with the ‘tree covering’ rather than ‘dog noise’ meaning). This way, interlocutors can maintain a common ground and avoid misunderstanding. Indeed, a recent encounter with a word-meaning can bias the later interpretation of the word towards this meaning (Rodd et al., 2016; Rodd, Lopez Cutrin, Kirsch, Millar, & Davis, 2013), suggesting that people update their word-meaning representations based on recent lexical usage. Although a considerable amount of research has investigated how information about *new* words and meanings is learned/consolidated, particularly over a 24-hour period involving sleep (e.g., Dumay & Gaskell, 2007), or even over a week (Tamminen & Gaskell, 2013), until recently relatively little work has focused on changes to the representations of *familiar* meanings of words (e.g., Fang & Perfetti, 2017). Those that do focus on familiar meanings (e.g., Rodd et al., 2013) tend to investigate the impact of encountering only one prior instance of an ambiguous word, thus it is unclear how word-meanings are updated by *multiple* recent encounters. For instance, recent encounters could have the same or different meanings and could be clustered or more spaced over time. The present experiments investigate how these different types of recent encounters may differentially affect the updating of word-meaning representations.

Accessing the meaning of a word is made challenging by the fact that over 80% of English words have multiple meanings (e.g., ‘bark’: the noise made by a dog or the covering of a tree; Rodd, Gaskell, & Marslen-Wilson, 2002). It has been shown that comprehenders make use of a range of cues to determine the most appropriate meaning of these semantically ambiguous words. These cues include the relative frequency with which a word-meaning occurs in a language (also known as meaning dominance) and the immediate sentence context in which the word is encountered. Much research has shown that the dominant (more frequently used) meaning is the default interpretation of the word unless immediate sentence context exists to steer interpretation towards a different meaning (e.g., Chen & Boland, 2008; Colbert-Getz & Cook, 2013; Foss, 1970; Rayner & Duffy, 1986; for an overview, see Vitello & Rodd, 2015). The use of meaning dominance reflects an optimal strategy in word interpretation on the part of the comprehender: when there is no cue to indicate otherwise, the listener is likely to interpret a word with its most frequent, “default” meaning. Such a view implies that people have representations of meaning frequencies that are relatively stable across time, as their default interpretation would only be overridden by immediate sentence context. For instance, the highly influential reordered access model takes both immediate context and long-term knowledge into account, but does not mention possible changes in word-meaning representations over intermediate time periods (Duffy, Morris, & Rayner, 1988).

However, the few recent studies on this topic confirm that recent linguistic experience can modulate, and can sometimes even overturn, the meaning dominance of an ambiguous word (Leinenger & Rayner, 2013; Poort, Warren, & Rodd, 2016; Rodd et al., 2013, 2016). Rodd et al. (2013) showed that when listeners encounter ambiguous words such as ‘fans’ without any biasing context, they are 30–40% more likely to interpret the words as referring to the subordinate (less common) ‘supporter’ meaning if they heard that subordinate meaning in a sentence (e.g., ‘the footballers were greeted warmly by the adoring *fans*’) 20 minutes earlier. Hence, just a single subordinate encounter increased the likelihood with which it was later used. This priming effect did not vary according to whether the same or a different voice was used for the prime sentence phase and the subsequent test phase, suggesting that word-meaning priming reflects an implicit updating of meaning frequencies in response to recent linguistic input rather than relying on episodic memories of the recently used meanings (Rodd et al., 2013, Experiment 2). Importantly, there was also evidence to suggest that this priming effect relied on repetition of the specific ambiguous word and was not driven by a more general form of semantic priming (Experiment 3): semantic priming from synonyms (e.g., supporter—fan) was evident at short prime-target delays (3 minutes) but was eliminated at the longer delays at which word-meaning priming has been studied (20 minutes or more). This finding is consistent with previous work showing that context alone (repetition of context *without* repetition of the ambiguous word per se) can affect later word interpretation over shorter prime-test intervals of a few minutes (Colbert-Getz & Cook, 2013).

The influence of a single word-meaning encounter on comprehension a few minutes later has been observed across different tasks (e.g., sentence reading, speeded lexical decision) and measures (e.g., eye tracking, EEG). Where context constrains the meaning of the ambiguous word at test, it is consistently shown that word-meaning comprehension is facilitated on a second encounter when the meaning is consistent with the first encounter (Binder & Morris, 1995, 2011; Copland, 2006). Again, encountering the ambiguous word itself is crucial to this comprehension facilitation, since reading subordinate context alone in a prime sentence (i.e., without the ambiguous word itself being presented) does not facilitate comprehension of the subordinate word-meaning itself when it is read up to a few minutes later (Leinenger & Rayner, 2013). Furthermore, comprehension can be (but is not always; Binder & Morris, 1995) impeded when the meaning of the second encounter is *inconsistent* with the first, showing that recent experience with a particular word-meaning can also hinder subsequent comprehension in cases where the subsequent encounter has the alternative meaning (Bainbridge, Lewandowsky, & Kirsner, 1993; Copland, 2006; Dholakia, Meade, & Coch, 2016; Simpson & Kang, 1994; Simpson & Kellas, 1989). Together, these very short-term (up to only a few minutes) priming studies clearly demonstrate that word-meaning representations are sensitive to very recent experience with those words, and can update rapidly to accommodate that experience.

In addition to these effects of prior experience with ambiguous words that occur within the timescale of a single experimental setting (up to 20 minutes), Rodd et al. (2016) show that if a person repeatedly uses/hears a word with its subordinate meaning over longer timescales of months or years, the meaning dominance for that word can be altered. For instance, rowers, who know additional rowing-related meanings for common English words (e.g., ‘feather’ and ‘square’ refer to positions of the oar), tend to interpret these words in light of their experience with these word-meanings even in non-rowing contexts. The tendency for rowers to interpret these words with rowing-related meanings increases with both additional years of rowing experience and decreased time since their last rowing practice. Converging evidence using ambiguous words which have additional baseball-related meanings shows that baseball experts, compared with non-experts, have more difficulty disambiguating sentences when they are strongly biased towards the non-baseball meaning (Wiley, George, & Rayner, 2016). Again, this shows a difficulty to disambiguate a word when the encountered meaning is inconsistent with one’s prior long-term experience. Taken together, these studies show that adults accumulate evidence across their life span to build lexical-semantic representations, using their linguistic experience across a range of timescales to guide interpretation.

This continual updating of word-meanings, driven by recent experience, plays a critical role in maintaining a common ground among interlocutors in language communication (Rodd et al., 2016) and in helping the listener to avoid misinterpreting a word and then having to engage in effortful reinterpretation processes (Rodd, Johnsrude, & Davis, 2010). It seems that interlocutors update their lexical-semantic representations based on their recent experience with the meanings of words. This allows comprehension to benefit from the most up-to-date likelihood of a particular meaning being the correct interpretation whenever an ambiguous word is encountered. Importantly, though, people seem to be able to capitalise on experience with words so that they can flexibly alter representations based on both longer-term (Rodd et al., 2016) and shorter-term (Rodd et al., 2013) experience. Unlike the view of stable lexical-semantic representations in adulthood, this dynamic “updating” approach suggests that adults’ comprehension is made more efficient by continuously learning from experiences with word meanings to make a “best guess” about the most likely intended meaning at any point in time.

But what is the mechanism that allows for word-meaning updating in response to recent experience? The finding that priming effects persist over 20–40 minutes in lab-based experiments (Rodd et al., 2016; Rodd et al., 2013) and several hours in more naturalistic settings (Rodd et al., 2016, Experiment 1) means that these changes in word-meaning availability are not easily accounted for by short-term priming mechanisms such as residual activation (e.g., Dell, 1986; McClelland & Rumelhart, 1981; Meyer & Schvaneveldt, 1971).

Similarly to the incremental learning account of repetition priming and semantic interference in speech production (Oppenheim, Dell, & Schwartz, 2010), Rodd et al. (2013) suggest that every encounter with an ambiguous word strengthens the connection between the word and the encountered meaning, such that experiences with word-meanings accumulate to enhance comprehension over time. More specifically, they proposed that the mechanism for the updating of word-meaning representations involves changes to connection strengths among units in a connectionist network (Rodd, Gaskell, & Marslen-Wilson, 2004), as this would allow transient changes in meaning availability to slowly accumulate across a life span. This learning mechanism, which has been proposed as an explanation for other types of long-term priming (e.g., Becker, Moscovitch, Behrmann, & Joordens, 1997), involves small but persistent changes to connection strengths between the relevant units within and/or across representational layers. For the updating of word-meaning representations, the changes to connection strengths reflect a build-up of evidence about the likelihood of a given meaning.

As for the relative likelihood of *different* meanings, if listeners continue to encounter both the dominant and subordinate meanings of a word, it is likely that they strengthen the relevant connections in proportion to the overall frequency with which each meaning is encountered, such that the availability of the different meanings reflects the relative frequencies of these encounters. For example, disambiguation of ‘bark’ could be influenced by recent encounters of *both* the ‘dog noise’ and ‘tree covering’ meanings. If an individual’s experience with a particular word changes systematically with time then, given sufficient experience, a previously subordinate meaning could eventually become the dominant meaning (which seems to be the case for the rowers reported in Rodd et al., 2016). As described by Rodd et al. (2013), connectionist models can accommodate this mechanism as long as they allow for updating/learning to continue throughout the model’s “lifespan”. In summary, it seems likely that repeated encounters with a word-meaning gradually strengthen the relevant connections in the lexical-semantic network and, over a relatively long period of time (e.g., months, years), can change an individual’s preferred meaning.

What is less clear is whether repeated encounters within a relatively *short* period of time (e.g., 20–30 minutes, compared with a lifetime of experience) can lead to similar cumulative effects in updating the representations of word-meanings. Changes in representation availability following a single encounter with a particular meaning do occur (Rodd et al., 2013; also see Bainbridge et al., 1993; Binder & Morris, 1995; Copland, 2006; Masson & Freedman, 1990, for comprehension facilitation from recent encounters in the space of a minute), but it is not known whether these relatively short-term changes in availability are sensitive to multiple, repeated encounters of a particular meaning within the same time-frame. It is also unclear whether repeated encounters of *different* meanings of an ambiguous word accumulate to have a combined effect on comprehension.

The repetition priming literature shows that multiple repetitions of words in a short space of time do increase the magnitude of priming compared with one repetition. This has been shown in lexical decision (Forbach, Stanners, & Hochhaus, 1974; Forster & Davis, 1984), word naming (Durso & Johnson, 1979), passage reading (Kolers, 1976), free recall, cued recall, and recognition (Nelson, 1977). A similar effect of repetition has been found in a test of explicit recall of words from a sentence, in which two presentations of an ambiguous word in a sentence improved recall compared to one presentation (Thios, 1972). However, this improvement was lessened when the second presentation used the alternative meaning of the ambiguous word, suggesting that encountering the dominant meaning interfered with the updated representation from an earlier encounter with the subordinate meaning. Together, these results indicate that multiple repetitions of an ambiguous word might lead to greater word-meaning priming than only one repetition, and that the effect of an initial exposure to a word meaning might be disrupted or abolished by a subsequent exposure to an alternative meaning of the word. However, the findings reported by Thios are in the explicit memory domain and therefore may be driven by different mechanisms than word-meaning priming (see Rodd et al., 2013), so it is not clear whether the repetition benefit and the interference from an alternative meaning would replicate in an implicit learning paradigm.

Given the repetition literature, it seems possible that multiple repetitions of an ambiguous word-meaning increase the likelihood of interpretation of the word towards that meaning compared to a single repetition. As argued above, this could occur through a process of cumulatively updating the relevant connection strengths within the lexical-semantic system upon each encounter with the word and meaning. However it is not clear whether the temporal spacing of these updates would further influence any such repetition benefit. That is, it remains unclear whether a particular temporal distribution of repetitions is most effective in changing the availability of word meanings: repetitions that are massed (i.e., temporally compressed), or repetitions that are spaced (i.e., temporally distributed). The existing literature shows inconsistent findings, such as no spacing benefit for cued recall (Greene, 1989), spacing benefit over massed for free recall (Madigan, 1969; Melton, 1970; Underwood, 1970), and no spacing benefit for free recall (Paivio, 1974). Multiple repetitions must at some level influence meaning availability over one repetition, otherwise the overall meaning dominance effect, (i.e., more frequent meanings being easier to access than less frequent meanings), and the increased availability of rowing meanings for rowers (Rodd et al., 2016), would not exist. Furthermore, if repetitions of different meanings are encountered then they might strengthen the relevant connections in proportion to the overall frequency with which each meaning is encountered, suggesting that a single subordinate followed by a single dominant repetition would both have an effect on how that word is later interpreted. Another possibility is that the relatively short-lived word-meaning priming effects, lasting, for example, 20–40 minutes, are solely driven by the most recent word-meaning priming encounter and that earlier encounters during this same timescale leave no (or minimal) trace. Under this view, the fact that the most recent encounter takes precedence over prior recent encounters would mean that changes to word-meaning preferences that occur over longer timescales (e.g., from days onward) would involve a different or additional learning mechanism, such as overnight consolidation.

The experiments reported here investigate, for the first time, whether and how recent repetitive encounters of ambiguous words in particular meaning contexts affect the availability of the primed meanings. Each of the three experiments followed the word-meaning priming paradigm first used by Rodd et al. (2013). Participants were exposed to repetitions of ambiguous words in subordinate meaning contexts and, after a filler task, these words appeared in a word association test to assess how the availability of the subordinate meaning had changed as a result of the prior exposure. This word association task, in which participants must comprehend a given word to respond with the first word that comes to mind, allows us to assess how ambiguous words are interpreted in the absence of the constraining semantic contexts that are used in tasks such as semantic relatedness judgments and thus provides a straightforward measure of participants’ default/preferred meanings. Broadly speaking, we assume that when participants provide an associate for a word, they first bring to mind one of the word’s meanings, and then report the first-generated associate of that meaning. Importantly, it does not seem to be the case that priming, as measured by word association, is driven purely by words remembered specifically from the prime sentence for an ambiguous word (items referred to as “primed associates”). That is, the priming effect does not rely on participants producing a response word at test that was encountered within the specific prime sentence (e.g., producing at test ‘footballers’ after being primed with ‘the footballers were greeted warmly by the adoring *fans*’), since removing these primed associates from the test data does not alter the pattern of priming (Rodd et al., 2013; Experiment 1). For these reasons, the word association test has become a commonly used method for assessing word-meaning priming and will therefore be used in the present experiments (Cai et al., 2017; Rodd et al., 2013, 2016).

In what follows we examine how multiple recent encounters with an ambiguous word, either in the same or a different meaning context, affect the later interpretation of these words (Experiment 1), and how this interpretation is influenced by the relative timing of multiple subordinate meaning repetitions (Experiments 2 and 3).

## Experiment 1

Experiment 1 had two aims. The first was to investigate whether multiple recent encounters with the *same* subordinate meaning boost the word-meaning priming effect compared with one encounter. Based on the mechanism for the updating of word-meaning representations proposed by Rodd et al. (2013) and Rodd et al. (2016), which assumes that the effects of multiple encounters with ambiguous words will accumulate over time, we predict that multiple subordinate repetitions presented within the same spoken paragraph (i.e., massed presentation) will boost meaning priming compared with one subordinate repetition. If this is the case, then it suggests that lexical-semantic representations are sensitive to the frequency of encounters during this time period and update cumulatively during this process.

The second aim was to examine the effects of encounters with *different* meanings of an ambiguous word. Specifically, we examine the case where the listener first encounters the subordinate meaning and then encounters the dominant meaning of the same word. The view that the effects of multiple encounters will accumulate over time predicts that both of these encounters will have an impact on subsequent disambiguation such that the dominant repetition will reduce the impact of the earlier exposure to the subordinate meaning. However, we also predict that there will still be a residual effect of the prior subordinate repetitions, compared to the case where only the dominant meaning is presented. If this were the case, then again it would support the view that lexical-semantic representations are updated in an incremental manner to reflect the relative frequency with which meanings occur.

**Figure 1 fig1:**
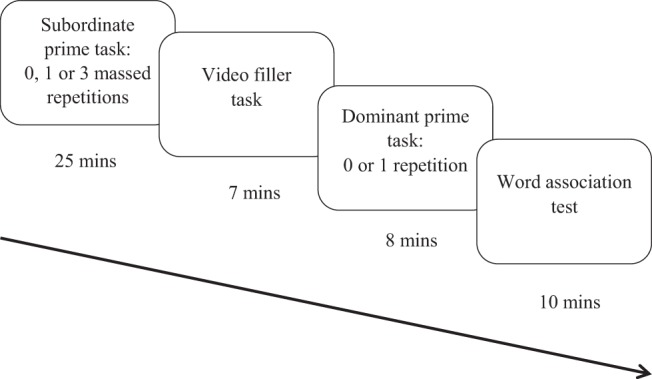
Experiment 1 task order, including prime phase elements, filler task, and test. The mean duration of each task is displayed below the figure.

This experiment used a modified version of the word-meaning priming paradigm developed by Rodd et al. (2013) with the addition of a dominant prime phase. That is, participants completed the subordinate prime phase, filler task, dominant prime phase and then a word association test phase (see Figure 1 for an overview of the procedure). In the subordinate prime phase, participants encountered a subset of the ambiguous words in the context of their subordinate meanings, either once or three times in massed presentation. The remaining (unprimed) ambiguous words were only presented during the test phase, which provided a baseline measure of meaning dominance for these items against which to compare the primed conditions. Hence, the prime phase involved three conditions: unprimed baseline, one repetition and three massed repetitions. After a filler task, which created a prime-test delay, participants encountered half of all words one more time, but in the context of their dominant meanings. Finally, in the word association test, participants heard all ambiguous words in isolation and responded with an associate, which provided a measure of each participant’s interpretation of the words. The mean length of the tasks resulted in an average delay between each item in the subordinate prime task and the word association task of approximately 30 minutes.[Fig-anchor fig1]

### Method

#### Participants

Thirty-three native British English speakers participated in the current experiment. However, only the data from 30 participants (23 females; mean age = 24.8, range = 18–40^1^) were analysed: one participant was excluded for exceeding age requirements and two participants were excluded because of a software error, which prevented task completion. All participants reported that they had no language, hearing or vision impairments (other than corrected-to-normal vision) and had lived in the U.K. for the majority of their lives, speaking English as their first language from birth. Participants were recruited via the University College London online recruitment system or advertisements on the university campus and paid the standard rate at the time of £6/hour^2^.

#### Materials

Sixty ambiguous words (e.g., bark, cabinet) were selected from a pretested set that had assessed dominance using a standard word association test (Warren, Vitello, Devlin & Rodd, in preparation; see the Appendix for ambiguous word list). These words had a dominance rating of 12–42% for the subordinate meaning (mean of 25%). In all cases the primed subordinate meaning had the same pronunciation and spelling as the dominant meaning, although in some cases there was an additional meaning with a different spelling (e.g., ‘break/brake’). Polysemous words were also included as long as the related meanings were judged by the author as sufficiently distinct that they could be distinguished on the basis of word association responses (e.g., typical associates related to the two related meanings of ‘wave,’ *disturbance in water* or *hand gesture*, were deemed sufficiently distinct, whereas those to the two meanings of ‘passage,’ *corridor/tunnel* or *journey over time/distance*, were not. Thirty-eight words were classed as polysemous; Parks, Ray, & Bland, 1998).

For the subordinate prime task, a total of 60 short paragraphs (mean length of 70 words) were composed in the style of a media or literature excerpt (see supplementary materials for paragraphs). Each paragraph contained at least one of the 60 ambiguous words, disambiguated towards the subordinate meaning^3^. For the three repetition condition, the ambiguous word was used in the paragraph three times and was therefore massed in presentation (i.e., the three repetitions appeared in quick succession, within the same paragraph). The first presentation of the word always occurred in the first sentence, with the second and third repetitions distributed throughout the remainder of the paragraph, e.g.,‘*The* ***cabinet*** *concluded that a referendum would be unnecessary, since the time it would use might only worsen the financial situation. The* ***cabinet*** *had been in talks for several weeks about a plethora of problems, but had only discussed the idea of a referendum over the last few days. Their decision was not a popular one, since previous* ***cabinets*** *held many referenda, which had proven popular with the public.’*

For the one repetition condition, the paragraphs were identical to the three repetition condition except that the second and third repetitions were replaced with a substitute word of a similar meaning. This was done to remove the instance of the ambiguous word itself without altering the global meaning or length of the paragraph. For example, the one repetition version of the passage above was created by replacing ‘*cabinet’*/‘*cabinets’* in the second and third sentence with ‘*politicians.’* To fully control the number of repetitions, the ambiguous words did not appear anywhere in the experiment except for their respective priming paragraphs and in the test task. The paragraphs were spoken by a British English speaker (Jennifer M. Rodd) and were digitally recorded in a sound-proof booth. For each paragraph, we created a written summary sentence (mean length 8.8 words), and participants rated how well this sentence summarised the paragraph (to encourage close attention to the paragraph; see Procedure). The summary for a given item was the same for both the one and three subordinate prime conditions. All summaries were designed to be a similarly reasonable level of quality (as quality-judgment/relatedness was the task for the participants, as explained in the Procedure).

These 60 ambiguous words formed the basis of the auditory word association test, with the addition of five unambiguous filler words that preceded these target items in the test. All words were recorded by the same female speaker as the prime paragraphs (see Rodd et al., 2013 for evidence that word-meaning priming is not dependent on, or enhanced by, consistency in speaker identity between prime and test).

Sixty sentences (mean length 9.2 words) were created for the dominant prime task. In each sentence, an ambiguous word was disambiguated towards the dominant meaning (e.g., ‘the cherry wood *cabinet* looked magnificent’), that is, a different meaning from in the subordinate prime test. These sentences were digitally recorded by a male speaker with a similar accent to the female speaker of the paragraphs. Each sentence was coupled with a written probe word that was either related (50%) or unrelated to its content (e.g., ‘furniture’; see Table 5 of supplementary materials for dominant-meaning sentences and probes).

A video animation (*Shaun the Sheep*, Aardman, 2010) was chosen as the filler task for several reasons. First, because controlling exposure to language is a key element to the word-meaning priming paradigm, this animation is ideal, as it does not involve any spoken or written words. Second, the content is not strongly related to any of the primed word meanings, and does not carry any strong emotional valence (strong valence stimuli were avoided for this task, as emotion can affect recall, e.g., Bock & Klinger, 1986; Cahill et al., 1996). Third, the animation is engaging for participants.

#### Design

This experiment had a within-subjects/between-item and within-item/between-subjects experimental design with two independent variables: subordinate meaning repetitions (3 levels: unprimed [no repetition], one repetition, three massed repetitions) and dominant meaning repetition (2 levels: unprimed [no repetition], one repetition). The dependent variable was the proportion of responses from the word association test were consistent with the *subordinate* meaning used in the priming paragraphs.

Each participant encountered each of the 6 conditions, with 10 items in each. The assignment of items to condition was rotated across six versions of the experiment, allowing each item to appear in only one priming condition for a given participant, yet across different participants, each item appeared in every priming condition. The number of items per condition and participant is shown in Table 1.[Table-anchor tbl1]

#### Procedure

The experiment was run in a cubicle, using the Qualtrics Inc. survey software (www.qualtrics.com). The experiment was displayed on a desktop computer but the video for the filler task was presented to participants on an Apple iPad. Participants wore headphones for the whole experiment to ensure that the stimuli could be heard easily and to minimise any background noise. Each participant was randomly assigned to one of the six versions of the experiment. After giving their informed consent, participants’ demographic data were collected and instructions for the experiment were displayed on screen. Trials within each task (subordinate prime task, dominant prime task, and word association) were randomised, each presented on a new page, with a mouse click (on-screen button) required to proceed to the next trial. Participants were given a practice trial and the chance to confirm instructions with the experimenter before each task. See Figure 1 for the sequence and timings of experimental tasks. To distract from the purpose of the experiment, participants were informed that they were taking part in two separate experiments. They were told that the “first experiment” (the subordinate prime task) was to pretest stimuli for another experiment and quality-check the summaries of the paragraphs, having been told that we were interested in their real opinion; the “second experiment,” they were told, consisted of watching a video and carrying out a filler task and then a final main task (in fact the dominant prime task and then the word association task, respectively).

##### Subordinate prime task

In each of 40 trials participants heard an excerpt, which included the ambiguous word in the context of the subordinate meaning, either once or three times, and saw the accompanying summary on-screen simultaneously. Participants were asked to rate on a five-point scale how well the summary sentence summarised the key information in the excerpt (1 = *poorly* to 5 = *excellently*).

##### Filler task

For the video animation, one of two selected episodes was played to participants (episode 1 length: 5 min, 55 seconds; episode 2 length: 5 min, 54 seconds). They were informed that they should pay attention to the content of the video, as they would be required to answer questions about it at the end of the experiment (although they were not asked questions, as this was only to disguise the aim of the experiment).

##### Dominant prime task

Participants subsequently completed the dominant prime task in which they were asked to listen to 30 sentences, each of which included an ambiguous word disambiguated towards the dominant meaning. For each sentence, they were asked to decide whether the sentence was semantically related to a probe word. The probe word was written on the screen during the sentence presentation, with ‘related’ and ‘unrelated’ buttons displayed. Although participants could respond before the end of the sentence, they were encouraged not to do so and to be as accurate as possible (participants were less likely to be accurate if they responded before sentence offset). This relatedness task was included to ensure that participants attended to the sentences and processed their meanings.

##### Word association test

Although the presentation order of experimental items in the word association test was randomised, the five filler items were always presented at the start of the test to get participants used to the nature of the task. Items were presented auditorily and participants were asked to type the first word they thought of when they heard each word into a textbox on the screen^4^. They were asked to type ‘0’ if they were unable to make out the word, unable to generate a response or felt uncomfortable giving one.

##### Post-experimental tasks

There were two tasks after the main experiment: awareness test and response-coding. For the awareness test, participants were asked two questions: ‘What do you think the aim of the experiments was?’ and ‘How many words from the word association do you recognise from the tasks earlier in the experiment?’ to measure awareness of the priming manipulation and investigate its impact on priming.

Participants were then asked to code their word association responses (blind to experimental condition) to clarify the meaning of each word that they had intended in their response. In this response-coding task, participants were presented with each word and their response. Provided with short definitions of the dominant and subordinate meanings of each item, they were asked to select to which meaning their response was related (or ‘other’ meaning), following the method of (Rodd et al., 2016). Finally, participants were debriefed and were given the opportunity to ask questions.

#### Task and coding checks

##### Subordinate prime task

All participants used the range of the 5-point scale for the summary ratings adequately indicating that they were engaged in comprehending the paragraphs (87% used the full range; those who did not use the full range did not rate any summaries as the lowest rating, which most likely reflects that the summaries were designed to be accurate). Summary rating means were consistent across subordinate prime conditions (one subordinate repetition *mean*: 3.56; *SD*: 1.25, three subordinate repetitions *mean*: 3.59; *SD*: 1.32).

##### Dominant prime task

All participants demonstrated accurate semantic relatedness judgments for the target words in this task (at least 80% correct responses), suggesting adequate engagement in the task.

##### Word association test

Responses were coded by participants as either (1) related to the dominant meaning of the homophone, (2) related to the subordinate meaning of the homophone, or (3) related to another meaning, ‘other’. To check that participants had coded responses correctly, the experimenter verified a 5% subset of coded responses. Since there were several incorrect codes, *all* coded responses (1s, 2s and 3s) were then verified by the experimenter by checking each code alongside the respective word association response. Any word association responses that were clearly associates of either the dominant or the subordinate meaning were recoded as such. For example, where participants coded their response ‘hot’ as ‘other meaning’ to the item ‘cold’ (presumably because it has the opposite meaning), their response was recoded as being related to the dominant (temperature) meaning by the experimenter. Because we were primarily interested in changes in the proportion of responses consistent with the primed subordinate meaning, for the analyses, ‘other’ responses (6%) were removed to provide a coded data set that indicated whether a participant gave a subordinate prime-consistent response or the dominant meaning of the ambiguous word.

### Results

#### Main analyses

**Figure 2 fig2:**
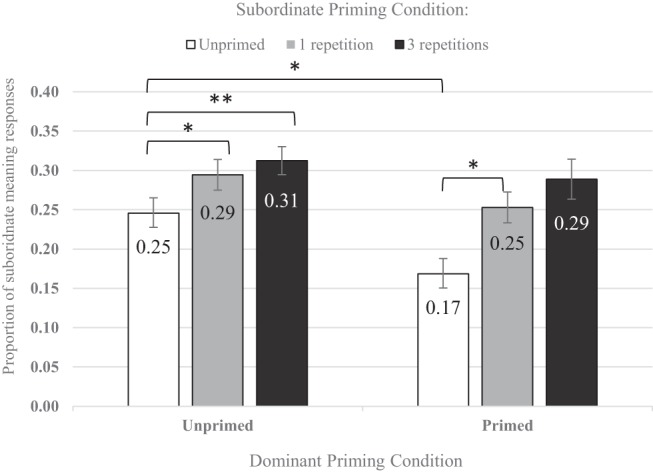
Experiment 1. Subject mean proportion of word association responses consistent with the primed subordinate meaning, with standard error bars adjusted for the within-subjects design.^5^ Significance level indicated with asterisks (* *p* < .05. ** *p* <.01) and simple effects shown for the theoretically important contrasts.

As is clear from the pattern of subject means in Figure 2, and as predicted, the subordinate priming increased the proportion of subordinate meaning responses, and the subsequent dominant priming reduced the proportion of subordinate responses. Interestingly, there seems to be little difference in priming between one and three subordinate repetitions.[Fig-anchor fig2]

The word association data were modelled using logistic mixed effects modelling (LMEM), with the glmer function from the lme4 package (version 1.1–7; Bates, Maechler, Bolker, & Walker, 2015) in R (version 3.3.1; R Core Team, 2016). LMEM is the most appropriate form of analysis for the present data since these data are binary, responses being subordinate *or not,* and this form of analysis takes the within-subject and within-item dependencies into account within a single model (Jaeger, 2008). As the subordinate meaning repetitions factor had three levels, we used two Helmert contrasts for this factor. These contrasts allowed for separate estimates of (a) the overall effect of subordinate priming (subordinate unprimed vs. the two subordinate repetition conditions combined) and (b) the effect of number of repetitions (one vs. three subordinate repetitions, omitting the unprimed control). Both factors were deviation coded for ease of interpretation of the model coefficients (subordinate repetitions contrast 1: unprimed = −2/3, one repetition = 1/3, three repetitions = 1/3; subordinate repetitions contrast 2: unprimed = 0, one repetition = −1/2, three repetitions = 1/2; dominant repetition: unprimed = −1/2, one repetition = 1/2).

A model was then built with five fixed effect coefficients (two to represent the subordinate meaning repetitions factor, as defined by the Helmert contrasts, one fixed effect for dominant meaning repetition, and two to represent the interaction between each of the subordinate meaning contrasts and the dominant factor) with a maximal random effects structure, as recommended to protect against inflated Type I error (Barr, Levy, Scheepers, & Tily, 2013). This full model failed to converge across all tests of main effects and interactions (most likely due to the complex random effects structure), so here and in subsequent experiments we followed the recommended protocol for dealing with nonconvergence (Barr et al., 2013). The random effects structure was simplified by removing one random effect term at a time (correlations removed first, then intercepts, then slopes^6^; the subject or item term that explained the least variance was removed first) until all of these nested models also converged. This resulted in the final model having a random effects structure comprising the subject and items intercepts only^7^. A model comparison approach (e.g., Baayen, Davidson, & Bates, 2008) was then used to determine the significance of the main effects of the subordinate and dominant meaning repetitions and their interaction. This approach involved individually removing the fixed factor of interest (e.g., the interaction term) and comparing it to the main model using a likelihood ratio test to examine whether the inclusion of the fixed factor of interest resulted in a significantly better model fit. Although the subordinate repetitions and interactions factors were each split into two by the Helmert contrast codes (see above for details), the two factors for each were either left in the model as a whole or removed as a whole for tests of the subordinate main effect and the interaction, respectively. In each case, a model without the fixed factor of interest was compared with the full model using a likelihood ratio test.

The main effect of subordinate repetitions was significant, χ^2^(2) = 16.64, *p* < .001, showing that there were more subordinate meaning word association responses following subordinate priming. The main effect of dominant repetition was also significant, χ^2^(1) = 6.68, *p* = .009, indicating that dominant priming reduced the number of subordinate meaning word association responses. However, the interaction between subordinate and dominant repetitions was not significant, χ^2^(2) = 1.71, *p* = .430, meaning that the interaction term did not significantly improve model fit compared with the model that only included the linear combination of the two predictors. This finding indicates that the reduction in subordinate meaning interpretations due to the dominant meaning encounter did not significantly vary as a function of the number of subordinate prime repetitions.

The overall significance of the subordinate repetitions factor appeared to be attributable to a significant difference between the subordinate primed and unprimed conditions; the model coefficient for the primed (both one and three subordinate repetitions) versus unprimed contrast was significant (β = 0.49, *SE* = 0.13, *z* = 3.87, *p* < .001), whereas the model coefficient for the one versus three repetitions contrast was not significant (β = 0.15, *SE* = 0.14, *z* = 1.12, *p* = .260). Pairwise comparisons with Tukey adjustment for multiple comparisons were conducted using the glht (general linear hypothesis testing) function in the multcomp package (version 1.4–1; Hothorn, Bretz, & Westfall, 2008). Comparisons confirmed that the one and three repetition conditions were both significantly different from the unprimed condition (β = −0.55, *SE* = 0.21, *z* = −2.54, *p* = .020 and β = −0.75, *SE* = 0.21, *z* = −3.58, *p* = .001, respectively).

To address questions about the significance of differences between specific conditions, we conducted a set of four simple effects analyses using subsets of the data, with Tukey-adjusted *p* values for post hoc comparisons. First, for subordinate unprimed *and* dominant primed words (i.e., words *not* presented during the subordinate prime phase but later presented during the dominant prime phase), there was a significant dominant priming effect where one dominant repetition increased the number of dominant word association responses compared to the unprimed baseline condition (which was subordinate *and* dominant unprimed; β = −0.52, *SE* = 0.21, *z* = −2.44, *p* = .010). This confirmed that the main effect of dominant repetitions was applicable to this particular simple effect comparison, demonstrating that, like with the subordinate meaning, a recent encounter with the *dominant* meaning of an ambiguous word biases the later interpretation of that word towards that same meaning, compared with when there is no recent encounter at all (i.e., the unprimed condition). Second, when words were primed with one subordinate repetition followed by one dominant repetition, this did not significantly alter word association responses compared to the unprimed baseline (β = 0.03, *SE* = 0.20, *z* = 0.14, *p* = .890). This result suggests that one subordinate meaning exposure shifts meaning preferences towards the subordinate meaning, and a subsequent exposure to the dominant meaning shifts meaning preferences back again, so that the effects of exposures to the two different meanings balance each other out. In other words, the combination of one subordinate and one dominant meaning exposure results in the returning of meaning preferences to a net level that is not significantly different to the unprimed baseline.

Most importantly, the combination of one subordinate and then one dominant repetition resulted in significantly more subordinate-meaning responses than exposure to one dominant repetition alone (β = −0.54, *SE* = 0.21, *z* = −2.50, *p* = .030). This shows that it is not only the most recent encounter that affects the priming-related shift in meaning preferences, but that an earlier encounter with an alternative meaning leaves a residual effect on preferences. However, the trend that three subordinate repetitions prior to the dominant repetition resulted in more subordinate meaning responses than one subordinate repetition prior to the dominant repetition was not significant (β = −0.21, *SE* = 0.20, *z* = −1.06, *p* = .540). This indicates that while an encounter with the subordinate meaning before exposure to the dominant meaning leaves a residual priming effect, three encounters with this subordinate meaning before the dominant meaning exposure do not increase this residual subordinate priming effect further.

#### Awareness checks

There were two awareness measures: awareness of experimental aim and awareness estimate, both of which were analysed with logistic mixed effects modelling to investigate their effect on priming. Two participants were removed because of missing data on the awareness test. One experimenter (Hannah N. Betts) coded the responses to the awareness of experimental aim question. If participants demonstrated some, or full, correct awareness of the experimental aim (e.g., ‘to see if the original sentences influenced my later associations’), their responses were coded as aware, whereas if they demonstrated little/incorrect or no awareness of the aim (e.g., ‘how large or small people’s semantic fields are’), their responses were coded as unaware, hence these data were dichotomous. Fifteen participants were unaware of the aim (priming effect across subordinate repetition conditions *mean* = .33, *SD* = .09) and 13 participants were fully/partially aware of the aim (priming effect *mean* = .27, *SD* = .07). The awareness estimate data were continuous, indicating participants’ estimates of the percentage of ambiguous words in the word association test that had been presented earlier in the experiment as a less explicit measure of awareness, (word estimate *median* = 33.5, *range* = 3–65, skewed distribution). These estimate data were rescaled (divided by 100) and centered.

Model comparisons^8^ revealed that neither the interaction between awareness of the experimental aim and subordinate priming, nor the interaction between the awareness estimate and subordinate priming, was significant, χ^2^(1) = 1.34, *p* = .248; χ^2^(1) = 0.16, *p* = .686, respectively, indicating that participants’ awareness of priming manipulation and how many test words were repeated from the prime phase did not influence subordinate meaning priming effects.

### Discussion

The aim of the present experiment was to investigate how multiple recent experiences with either the same or different meanings of an ambiguous word affect subsequent disambiguation. Just one encounter with the subordinate meaning of an ambiguous word was sufficient to retune lexical-semantic representations 30 minutes later, thus replicating previous findings (Rodd et al., 2013, 2016). A single encounter with an ambiguous word in the context of its subordinate meaning resulted in a significant increase in the proportion of responses consistent with this meaning, compared to the unprimed baseline. The average dominance of the primed subordinate meanings increased from a baseline of 25% to 29%, showing that although these subordinate meanings are, on average, still less preferred than the alternative dominant meaning, they are more readily available following recent exposure. Although there was a numerical effect suggesting that unaware participants showed a larger subordinate priming effect, analyses showed that this was not significant. Whilst it is reassuring that awareness of priming did not significantly alter subordinate priming, Experiments 2 and 3 will follow up on these awareness analyses with more participants and therefore more power.

Although both the one and three massed subordinate repetition conditions significantly shifted disambiguation towards the subordinate meaning compared to the baseline (*relative* increases of 16% and 24%, respectively), three massed subordinate repetitions did not provide a significant additional biasing effect over and above one repetition of the subordinate meaning. In contrast to the mechanism proposed by Rodd et al. (2013) whereby every encounter with an ambiguous word produces a similar change to connection strengths, the present experiment finds no evidence to support the notion that each encounter with an ambiguous word increases the availability of the primed meaning to the same extent, at least when these encounters occur within a single paragraph (i.e., massed presentation).

One encounter with the dominant meaning was also sufficient to retune representations. This finding contradicts the predictions of the literature (Rodd et al., 2013, Experiment 1, Figure 1b), which suggests that there would be little effect of dominant priming since the dominant meaning is already the most available meaning and therefore cannot be made much more available. However, the delay between the dominant prime phase and test is markedly shorter than the delay between the subordinate prime phase and test, which could account for the dominant priming effect and makes it difficult to compare the magnitudes of dominant and subordinate meaning priming.

Importantly, as predicted, there was still an observable effect of prior subordinate meaning repetitions following the dominant repetition: there were significantly more subordinate meaning responses when a word was primed with the subordinate and then dominant meaning, compared to priming the dominant meaning alone. In other words, prior subordinate priming has a residual effect that persists after exposure to the dominant meaning. Interestingly, one subordinate exposure followed by one dominant exposure was comparable to the unprimed baseline condition, with the effects of the two “opposite direction” manipulations effectively cancelling each other out. Clearly, it is not the case that only the most recently activated meaning drives subsequent disambiguation. Instead, at least in the case where *different* meanings of a word are encountered with a substantial (23.5 minute) gap between the encounters, disambiguation seems to reflect a cumulative effect of recent experiences.

In contrast to this cumulative effect for encounters with different meanings of a word, this experiment found no evidence that multiple recent encounters with the *same* (subordinate) meaning can produce a significantly greater biasing effect compared with just one encounter. This finding is surprising: multiple repetitions must at some level influence disambiguation over and above the effect of one repetition, otherwise there would be no effect of relative meaning frequencies on word interpretation, nor would there be an effect of an individual’s long-term experience with word meanings, ranging from hours to years (Rodd et al., 2016). Why, then, in the present experiment did multiple repetitions not significantly boost availability of the subordinate meaning any more than one repetition?

One possibility is that, in the one repetition condition, the substitute words that were used in place of the second and third repetitions caused participants to reactivate the initial ambiguous word such that the priming effect in the one repetition condition was artificially inflated. Any semantic priming resulting from these substitute words is not likely to persist at a 30-minute delay (Rodd et al., 2013), so this account would have to assume that the ambiguous word itself was covertly reactivated. Another possibility is that it is the massed presentation of the multiple repetitions within single paragraphs that could explain the absence of any additional priming boost, and perhaps spacing these repetitions would increase priming compared with the single exposure condition. Indeed, for the condition in which participants encountered the subordinate and then the dominant meaning (where there is evidence of cumulative effects of multiple encounters), these encounters were spaced. The repetition priming literature provides some evidence to suggest that spacing might indeed boost priming (Glenberg, 1976; Greene, 1989; Madigan, 1969; Thios, 1972; Underwood, 1970), although not necessarily (Paivio, 1974). More specifically, the natural language processing literature suggests a “One Sense per Discourse” principle (e.g., Gale, Church, & Yarowsky, 1992) where an ambiguous word appearing multiple times within a discourse has a high (up to 98%) chance of each repetition having the same meaning. As a result, within-discourse repetition is most likely to (overall) provide *one* piece of information about only *one* meaning regardless of how many repetitions are encountered and is therefore unlikely to be representative of a wider language context. This within-discourse repetition would be less informative for improving future interpretation than between-discourse repetitions, which have multiple different contexts and would therefore provide *multiple* pieces of evidence about *one* meaning. Hence one or three subordinate repetition(s) within the same discourse (i.e., paragraph) would not lead to different levels of priming. In light of these possibilities, we further investigated the nature of multiple repetitions in Experiment 2.

## Experiment 2

**Figure 3 fig3:**
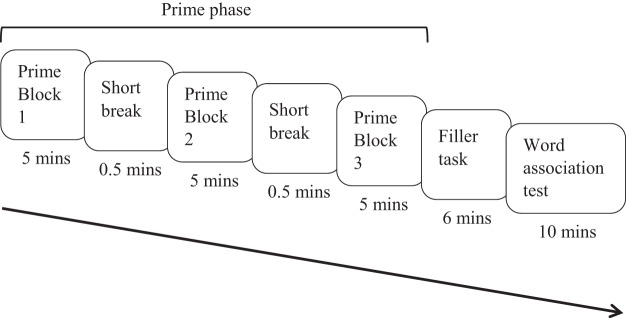
Experiment 2 task order, including prime phase elements, filler task, and test. The mean duration of each task is displayed below the figure.

This experiment used single sentence primes rather than paragraphs to allow for the temporal spacing of repetitions (as in Rodd et al., 2016, Experiment 2; Rodd et al., 2013). The prime phase was divided into three blocks to allow for the three repetitions of an ambiguous word (each in a different sentence) to be spaced across the prime phase (i.e., one repetition in each block). We compared the word-meaning priming effect between these three spaced repetitions with that of one repetition, where the ambiguous word was only encountered once in the prime phrase. To ensure that any benefit seen in the spaced repetition condition over the one repetition condition did not arise as a result of primacy or recency effects (i.e., greater priming for words encountered either early or late in the experiment), two ‘one repetition’ conditions were included: an early repetition condition, where the ambiguous word appeared in the first prime block, and a late repetition condition, where the ambiguous word appeared in the third prime block. Unlike Experiment 1, we did not include a dominant meaning priming manipulation. Hence, the experiment had four conditions: unprimed baseline, one early repetition (block 1), one late repetition (block 3) and three spaced repetitions (one repetition in each of blocks 1, 2 and 3). This subordinate meaning prime phrase was followed by a filler task, which created a prime-test delay, and then by a word association task, where participants heard all ambiguous words in isolation and responded with an associate. See Figure 3 for an overview of the procedure.[Fig-anchor fig3]

### Method

#### Participants

Sixty-four native British English speakers participated in the current experiment, although only the data from 55 participants (38 females; mean age = 21.5, range = 18–33) were analysed. The data from three participants did not save because of a technical issue and six participants were excluded for not meeting the eligibility requirements. All remaining participants met the requirements specified in Experiment 1 and were recruited in the same way but were paid the standard rate at the time of £8/hour.

#### Materials

The 88 ambiguous words were taken from Rodd et al. (2016, Experiment 2). These words were chosen to have a subordinate meaning that was semantically distinct from the dominant meaning (dominance range of the subordinate meanings = 0–0.48, *M* = 0.24). Forty-nine (56%) of these ambiguous words had also been used in Experiment 1 (see the Appendix for full word list). As with Experiment 1, polysemous words were also included as long as the related meanings were judged by Hannah N. Betts as sufficiently distinct that they could be distinguished on the basis of word association responses (this accounted for 50 words; Parks et al., 1998).

For the subordinate prime task, there were three sentences constructed for each of the 88 ambiguous words (mean length = 9 words; one sentence for each word was used in Rodd et al., 2016, Experiment 2). All three sentences disambiguated the word towards the same subordinate meaning but with different contextual details (see Table 2 for an example). This ensured that the multiple repetitions only primed the meaning of the word and not the entire sentence. Disambiguating context always preceded the ambiguous word so that, upon encountering the homophone, only the intended subordinate meaning was appropriate. Each sentence was coupled with a probe word, which was either related or unrelated in meaning to the sentence (unrelated probes were not related to any meaning of the ambiguous word; see supplementary materials for sentences and probe words). The relatedness of probes was assigned at random to each sentence, although within each set of three sentences per ambiguous word, at least one probe was related and at least one was unrelated. Across the set of items, 50% of probe words were related. The target ambiguous words did not appear in any other sentences, instructions, or other tasks, or as any of the probe words throughout the experiment. Sentences and probe words were presented in auditory form and spoken by a female native British English speaker with a Southern English accent (Hannah N. Betts).[Table-anchor tbl2]

The 88 experimental ambiguous words were all included in the word association test, together with a further 20 unambiguous filler words, which were included to reduce the proportion of primed ambiguous words in the task with the aim of making the prime manipulation less salient. The first four ambiguous words in this task were filler ambiguous words, to allow participants to become accustomed to the task. All words were presented auditorily, in the same voice as the prime sentences. As with Experiment 1, a video animation (*Shaun the Sheep*, Aardman Animations Ltd., 2010) was chosen as the filler task (see Experiment 1 for details).

#### Design

This experiment had a within-subjects design where all participants encountered all conditions but with a different set of items in each condition, so that each item appeared in every condition across participants. There was a single factor, subordinate prime repetitions, which had four levels: unprimed, one early repetition, one late repetition and three spaced repetitions. The dependent variable was the number of word association responses consistent with the primed subordinate meaning.

In the subordinate prime task there were three experimental blocks (see Figure 3). Participants encountered 22 ambiguous words in the first experimental block that were assigned to the one early repetition condition, 22 ambiguous words in the third experimental block that were in the one late repetition condition, and 22 ambiguous words in the three spaced repetition condition, which had one repetition in each of the three blocks. Participants therefore encountered 66 experimental sentences in total in the prime phase. To achieve an equal number of sentences in each block, 22 unambiguous fillers were added to block 2 for a total of 44 sentences per block. There were five additional unambiguous filler sentences presented at the start of each experimental block. Finally, 22 ambiguous words were assigned to the unprimed condition and thus were not encountered in the prime phase, but were presented in the word association test to provide an unprimed baseline proportion of subordinate meaning responses.

Four versions of the experiment were created so that each ambiguous word appeared in each condition but for different participants, ensuring that participants saw each ambiguous word in only one condition. Thus, all ambiguous words and all participants contributed to all conditions. Within each version, three subversions were created, since there were three sentences for each ambiguous word but only one of which would be displayed in the one repetition condition. In the multiple repetition condition, participants saw all three sentences for each ambiguous word, but the order of these three sentences varied across participants in different subversions. In the single repetition condition, across participants, a different sentence of the three was presented, rotated across subversions, to control for any potential differences between the three sentences.

#### Procedure

The experiment was presented using MATLAB R2013b (version 8.2.0.701). All details regarding experiment set-up and preparation (e.g., demographics and instructions) were identical to Experiment 1 with the exceptions of a key press being required to proceed to the next screen or trial (as opposed to the mouse click in Experiment 1), and here the filler video was presented on the same screen as the other tasks (rather than via an iPad). See Figure 3 for a summary of the sequence and timings of the tasks.

Across all conditions there was an average delay of approximately 19 minutes between an ambiguous word in the subordinate meaning prime task and the same ambiguous word in the word association task. The average delays between an ambiguous word in block one and block three of the prime task and the same word in the word association task were 13.5 minutes and 24.5 minutes, respectively. Hence, there was an 11-minute average difference between the one early repetition and one late repetition conditions.

##### Subordinate prime task

Participants heard each sentence and, upon sentence offset, saw the probe word on-screen and were asked to respond as quickly and accurately as possible to the probe by either pressing the ‘r’ key for related or the ‘u’ key for unrelated. Response times longer than 3 seconds prompted a message encouraging faster responses on subsequent trials. The key press response triggered the next trial. There was a 30 second break for participants between each of the three experimental blocks. Five filler trials started each block, with the remaining items presented in a random order after the initial filler trials. The fillers at the start of each block were included to prevent the possibility that two of the spaced sentences for the same ambiguous word were encountered in close proximity, that is, at the very end of one block and then at the very start of the subsequent block.

##### Filler task

The filler task involved watching a video animation (see Experiment 1 Procedure for details).

##### Word association test

The procedure was the same as that used in Experiment 1, with the addition of a message encouraging faster responses on subsequent trials when the time to first key press exceeded 3 seconds.

##### Post-experimental tasks

The awareness questions were the same as those used in Experiment 1. Participant self-coding was not used in this experiment, or in Experiment 3, as the quality of participant coding in Experiment 1 was low and therefore required recoding by an experimenter (Hannah N. Betts).

#### Task checks and coding

All participants had at least 75% accuracy on the semantic relatedness task, suggesting adequate engagement in the subordinate meaning prime task.

There were two coders (Hannah N. Betts and a research assistant) for the word association response data and coders were blind to the condition. Each word association response was coded either as being related to (1) the dominant meaning, (2) the primed subordinate meaning, (3) ‘other’, which included alternative meanings of the word, responses which were ambiguous/unclear and ‘0’ responses (which participants were instructed to give if they could not think of a response or felt uncomfortable giving a response). For example, for the subordinate meaning of ‘glasses’ as in the sentence ‘she poured the champagne into the glasses,’ the word association response ‘eyes’ would indicate the dominant meaning, whereas the response ‘drink’ would indicate the primed, subordinate meaning. Each experimenter coded half of the data. Any uncertainties were discussed with another author (Jennifer M. Rodd) and if any doubt remained as to which meaning a participant intended, the response was coded as ‘other’. For the analyses, ‘other’ responses (10%) were removed, as in Experiment 1.

### Results

#### Main analyses

**Figure 4 fig4:**
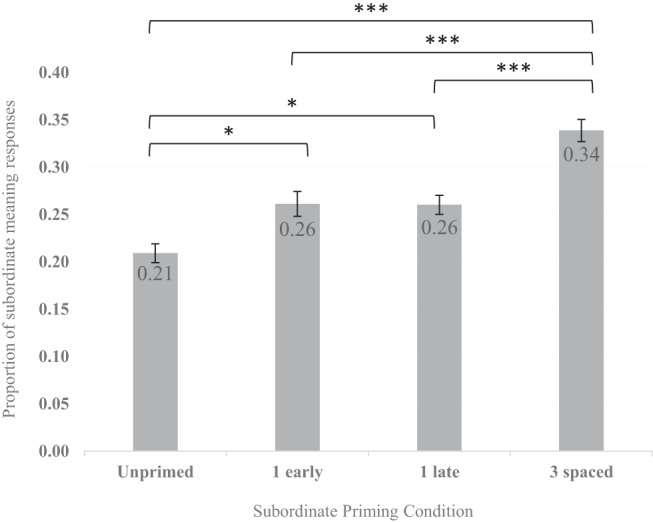
Experiment 2. Subject mean proportion of word association responses consistent with the primed subordinate meaning, with standard error bars adjusted for the within-subjects design and significance level indicated with asterisks (* *p* < .05. *** *p* <.001).

As the subject means in Figure 4 indicate, relative to the unprimed condition, the proportion of subordinate responses increased following one repetition of the subordinate meaning, and increased again following three spaced repetitions.[Fig-anchor fig4]

As with Experiment 1, a model with a maximal random effects structure was built with a fixed effect for subordinate meaning repetitions. The full model failed to converge. Following the recommended protocol for this issue (see Experiment 1 analyses for details; Barr et al., 2013), the correlations between the intercepts and slopes for subjects and items were removed, allowing the model to converge.

The model comparisons revealed a significant main effect of subordinate meaning repetitions, χ^2^(3) = 69.60, *p* < .001, indicating that responses to ambiguous words varied as a function of the number of subordinate meaning repetitions encountered in the prime task. Pairwise comparisons with Tukey adjustment compared each level of the repetitions factor (unprimed baseline, one early repetition, one late repetition, three spaced repetitions) with one another (adjusted *p* values reported). Comparisons revealed significantly more subordinate prime-consistent responses following one early repetition (β = −0.38, *SE* = 0.15, *z* = −2.50, *p* = .050), and following one late repetition (β = −0.38, *SE* = 0.14, *z* = −2.70, *p* = .030), compared to the unprimed baseline. However, there was no significant difference between the single early and late repetitions (β = 0.002, *SE* = 0.13, *z* = 0.01, *p* = .990). Importantly, there were significantly more subordinate prime-consistent responses following three spaced repetitions than the one early repetition condition (β = 0.49, *SE* = 0.12, *z* = 4.06, *p* < .001), one late repetition condition (β = 0.49, *SE* = 0.12, *z* = 4.13, *p* < .001) and the unprimed baseline (β = −0.88, *SE* = 0.13, *z* = −6.71, *p* < .001).

#### Awareness checks

The two awareness measures, awareness of experimental aim and awareness estimate, were analysed with logistic mixed effects modelling to investigate their effect on priming as outlined in Experiment 1. Two participants were removed due to missing data on the awareness test. Twenty-eight participants were unaware of the aim (priming effect across subordinate repetition conditions *mean* = .28, *SD* = .05) and 25 participants were fully/partially aware of the aim (priming effect *mean* = .30, *SD* = .05), where the word estimate gave an overall implicit measure of awareness (*median* = 60, *range* = 0–150, skewed distribution).

Model comparisons^9^ revealed that neither the interaction between awareness of the experimental aim and subordinate priming, nor the interaction between the awareness estimate and subordinate priming, was significant, χ^2^(1) = 1.34, *p* = .247; χ^2^(1) = 0.002, *p* = .967, respectively), indicating that participants’ awareness of the priming manipulation and how many test words were repeated from the prime phase did not influence subordinate meaning priming effects.

### Discussion

The aim of Experiment 2 was to investigate the impact of spacing repetitions of a word-meaning to see how multiple recent experiences with the same meaning affect how that word is later interpreted. First, the results indicate that just one encounter with the subordinate meaning of an ambiguous word can influence how that word is disambiguated approximately 19 minutes later. This word-meaning priming effect replicates the corresponding comparison from Experiment 1 (subordinate one repetition vs. subordinate unprimed, without dominant meaning priming) as well as previous findings (Rodd et al., 2013, 2016). Moreover, awareness analyses supported findings from Experiment 1 that awareness does not significantly alter priming, where Experiment 2 showed a smaller and still non-significant numerical increase in subordinate priming for unaware participants than Experiment 1.

Second, the meaning priming effects for the early and late single repetition conditions did not significantly differ. The average time difference between these conditions was 10 minutes, hence a 24-minute prime-test delay for the early repetition condition and a 14-minute prime-test delay in the late repetition condition. This is consistent with previous findings: after a rapid decline during the first few minutes, word-meaning priming effects seem relatively stable across this time window (Rodd et al., 2016, Experiment 2). While the prime-test delay for the late condition was less than the 19-minute delay used by Rodd et al. (2013), which showed that semantic priming did not persist, the similarity in priming effects from the early and late conditions is in contrast to what would be expected if the late condition were advantaged by semantic priming additionally to meaning priming. Furthermore, we would suggest semantic priming is unlikely given that it is generally short-lived, where an effect is considered ‘long-term’ if it survives a few minutes and intervening items (Becker et al., 1997).

Third, repeating the same subordinate word-meaning three times, spaced over the prime phase, increased the priming effect beyond that of one repetition. Compared to the unprimed baseline, one repetition provided a relative increase in the number of subordinate meaning preferences of 24%, whereas three spaced repetitions provided a more substantial relative increase of 62%. As there was no significant difference between the early and late one repetition conditions, it seems that there was no presence of a primacy or recency effect (from an encounter in the first or third prime block, respectively) and hence the benefit of spacing is not simply attributable to the spaced condition consistently containing a prime in the first and last block, but is instead attributable to the multiple spaced repetitions themselves. This benefit of spaced repetitions shows that, at least in some cases, multiple individual encounters with an ambiguous word in a particular meaning context might further strengthen the relevant connections in the lexical-semantic network, producing a greater biasing effect over a single encounter (Rodd et al., 2013). This is consistent with the findings by Thios (1972) that spacing of repetitions improves task performance (recall of words in a sentence) compared to massed and single presentations.

Although the present findings suggest that the absence of a priming boost following three repetitions in Experiment 1 was attributable to their massed nature, these two experiments differ in several ways other than the spacing of the ambiguous words. Most notably this experiment used separate unrelated sentences and not connected paragraphs as in Experiment 1. Therefore, to be sure that it is the spacing of the ambiguous words that is key to determining the presence/absence of a boost in priming for multiple repetitions relative to one repetition, the three massed and three spaced repetition conditions need to be directly compared in the same experiment using the single sentence stimuli. Experiment 3 will therefore directly compare one repetition, three massed repetitions and three spaced repetitions in their word-meaning priming effects.

## Experiment 3

**Figure 5 fig5:**
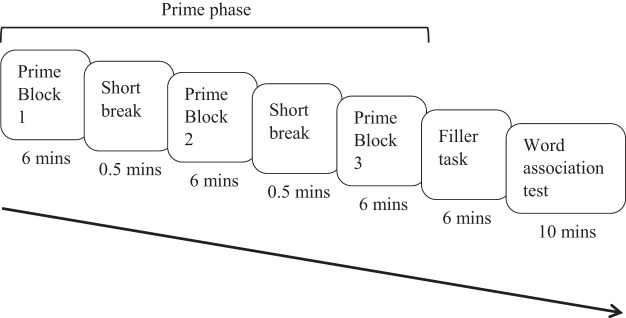
Experiment 3 task order, including prime phase elements, filler task, and test. The mean duration of each task is displayed below the figure.

This experiment includes four conditions: unprimed baseline, one repetition, three massed repetitions, and three spaced repetitions. As in Experiment 2, the three spaced repetitions were spread across the three blocks of the prime phase, with one sentence per block. The three massed repetition sentences were presented as consecutive sentences within the same (randomly selected) block. The one repetition sentences were also distributed randomly across the three blocks. Because block position did not affect the magnitude of priming in Experiment 2, we did not counterbalance the block position in the one repetition condition. After the filler task, participants heard all ambiguous words in isolation and responded with an associate as a measure of their interpretation of the ambiguous word. See Figure 5 for an overview of the procedure.[Fig-anchor fig5]

### Method

#### Participants

Sixty-one native British English speakers participated in the current experiment. Three participants were excluded for not meeting the eligibility requirements (see Experiment 1) and the remaining 58 participants (46 females; mean age = 20, range = 18–32) were entered into the analyses. Participants were paid the standard rate at the time of £8/hour.

#### Materials

See Experiment 2 Materials for details. The materials used in the current experiment are identical; only the design differed.

#### Design

In a within-subjects/between-item and within-item/between-subjects experimental design, the independent variable was the number of subordinate prime repetitions, which had four levels: unprimed, one repetition, three massed repetitions, and three spaced repetitions. The dependent variable was the number of word association responses consistent with the primed subordinate meaning.

In each version, 22 of the total 88 ambiguous words were included in each of the four conditions. The 22 items in the one repetition condition and the 22 three-sentence sets in the massed repetition conditions were distributed across the three experimental blocks (for each of these two conditions: 8 items in block 1, 7 items in block 2, 7 items in block 3), whereas for the 22 spaced repetition items, one sentence per item was allocated to each block. For each participant there were 22 ambiguous words that were not encountered in the prime phase but were included in the word association test to act as an unprimed baseline.

Four versions of the experiment were created so that each ambiguous word appeared in each condition but for different participants, ensuring that participants saw each ambiguous word in only one condition.

#### Procedure

The general procedure used in the current experiment is the same as in Experiment 2; only the design of the repetition differed. As the inclusion of the massed condition involved two additional sentences per item (compared with the single repetition conditions in Experiment 2), the prime phase was longer (timings shown in Figure 5): the average delay between prime and test encounters increased from 19 minutes in Experiment 2 to 21 minutes here.

The sets of three sentences that were presented in the massed and spaced conditions were always presented in the same order (the order of the three sentences was randomised following creation of the sentences). For the one repetition condition, one of the three sentences was randomly selected for each participant.

#### Task checks and coding

All participants had at least 75% accuracy on the semantic relatedness task, indicating adequate engagement in the prime task.

For the word association test responses the coding scheme was the same as for Experiment 2. One coder (Zainab B. Okedara) completed all response coding, a subset of which was then verified by the second coder (Hannah N. Betts). Any uncertainties were discussed with another author (Jennifer M. Rodd) and if any doubt remained as to which meaning a participant intended, the response was coded as ‘other’. The item ‘cold’ was excluded from all analyses as there were too many responses coded as ‘other’ (28 out of 61), reflecting the fact that many common responses were indistinguishable between the ‘temperature’ and ‘illness’ meanings. For the analyses, ‘other’ responses (11%) were removed, as in Experiment 1.

### Results

#### Main analyses

**Figure 6 fig6:**
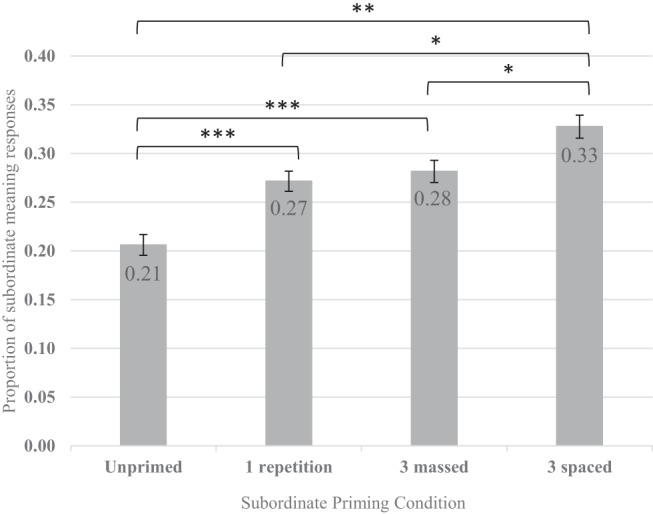
Experiment 3. Subject mean proportions of word association responses consistent with the primed subordinate meaning, with standard error bars adjusted for the within-subjects design and significance level indicated with asterisks (* *p* < .05. ** *p* <.01. *** *p* <.001).

As the subject means in Figure 6 indicate, the proportion of subordinate responses increased following both one repetition and three massed repetitions of the subordinate meaning, relative to the unprimed condition. There was a further increase following three spaced repetitions.[Fig-anchor fig6]

As with Experiments 1 and 2, a model with a maximal random effects structure was built (Barr et al., 2013) with a fixed effect for subordinate meaning repetitions and random effects for subjects and items. The full model failed to converge so the random effects structure was progressively simplified until the model converged, resulting in an intercepts-only random effects structure.

As with Experiment 2, a model comparison approach revealed a significant main effect of subordinate meaning repetitions, χ^2^(3) = 58.7, *p* < .001, indicating that responses to ambiguous words varied as a function of the number of subordinate meaning repetitions in the prime task. Pairwise comparisons with Tukey adjustment compared each level of the repetitions factor (unprimed baseline, one repetition, three massed repetitions, three spaced repetitions) with one another (adjusted *p* values reported). Comparisons revealed significantly more subordinate prime-consistent responses following one repetition compared to the unprimed baseline (β = −0.45, *SE* = 0.11, *z* = −4.23, *p* < .001). There were also significantly more subordinate responses following three massed repetitions compared to the unprimed baseline (β = −0.53, *SE* = 0.11, *z* = −4.96, *p* < .001), but no significant difference between the one repetition and three massed repetition conditions (β = −0.08, *SE* = 0.10, *z* = −0.80, *p* = .880). Critically, there were significantly more subordinate responses following three spaced repetitions compared with all other conditions: three massed repetitions (β = 0.26, *SE* = 0.09, *z* = 2.62, *p* = .040), one repetition (β = 0.34, *SE* = 0.10, *z* = 3.37, *p* = .004) and the unprimed baseline (β = −0.80, *SE* = 0.10, *z* = −7.53, *p* < .001).

#### Awareness checks

The two awareness measures, awareness of experimental aim and awareness estimate, were prepared for logistic mixed effects modelling to investigate their effect on priming as outlined in Experiment 1. One participant was removed because of missing data on the awareness test. Thirty-one participants were unaware of the aim (priming effect *mean* = 0.27, *SD* = 0.05) and 29 participants were fully/partially aware of the aim (priming effect *mean* = 0.28, *SD* = 0.05), where the word estimate gave an overall implicit measure of awareness (*median* = 50, *range* = 1–100, skewed distribution).

Model comparisons^10^ revealed that neither the interaction between awareness of the experimental aim and subordinate priming, nor the interaction between the awareness estimate and subordinate priming, was significant, χ^2^(1) = 0.01, *p* = .923; χ^2^(1) = 1.15, *p* = .282, respectively, indicating that participants’ awareness of the priming manipulation and how many test words were from the prime phase did not influence subordinate meaning priming effects.

### Discussion

The aim of the present experiment was to investigate the impact of spacing priming encounters to see how recent experiences with a particular meaning of an ambiguous word affect subsequent disambiguation. As with Experiments 1 and 2, just one encounter with the subordinate meaning of an ambiguous word influenced how that word was disambiguated approximately 21 minutes later: there was a 29% relative increase in the proportion of subordinate responses from the unprimed to the one repetition condition, thus replicating the word-meaning priming effect (Rodd et al., 2013, 2016). Moreover, awareness analyses supported findings from Experiments 1 and 2 that awareness does not significantly alter priming, despite a small numerical effect in the opposite direction to Experiments 1 and 2 suggesting a non-significant *decrease* in subordinate priming for unaware participants.

As in Experiment 1, the magnitude of the word-meaning priming effect did not significantly increase following three massed presentations of sentences with the subordinate meaning compared to the condition with only one priming sentence. In contrast, priming did significantly increase when the three sentence presentations were spaced, resulting in a sizeable 22% *relative* increase compared to the one repetition condition. Critically, spaced repetitions also significantly increased the priming effect compared to massed repetitions with the same number of sentences (an 18% relative increase). It seems that when multiple repetitions occur in quick succession they act similarly to a single instance, and it is not until those repetitions are separated that there is an additional effect of multiple encounters with the word and its subordinate meaning. Hence, it seems that the spacing of experiences with ambiguous words is key to producing greater alterations to the lexical-semantic network than that of one experience.

## General Discussion

The aim of the current experiments was to explore how listeners update their lexical-semantic knowledge on the basis of experience. Specifically, using a contextual prime and word association test paradigm, three experiments investigated how single and multiple experiences with ambiguous word-meanings influence the later interpretation of these words in isolation. The results can be grouped into three main findings.

### Effects of Single Subordinate and Dominant Encounters

All three experiments show that a single encounter with a subordinate word-meaning was sufficient to bias how that word was interpreted when presented in isolation after a 20–30 minute delay. These findings replicate four experiments from the literature (Rodd et al., 2016, Experiments 1 & 2; Rodd et al., 2013, Experiments 1 & 3), providing a total of 7 experiments that have consistently shown this robust word-meaning priming effect within the subordinate prime/word association test paradigm. These experiments also replicate the finding that participants’ awareness of the experimental aims is not a critical factor for priming to occur. In all three experiments, there was no significant interaction between the magnitude of priming and participants’ awareness of the experimental manipulation. Further, the numerical effects of awareness on priming were inconsistent across experiments: while in Experiments 1 and 2 we observed (non-significantly) more priming for the ‘unaware’ participants compared to the ‘aware’ participants, for Experiment 3 we observed the reverse (non-significant) effect. This suggests that the word-meaning priming observed in this paradigm is *not* driven by conscious attempts to recall previous sentences.

Experiment 1 goes beyond this replication; whereas previous studies of word-meaning priming have focused on the situation where participants are primed with the subordinate (less frequent) meaning, we observed, for the first time, a significant effect of prior experience with the word’s *dominant* meaning. Although the dominant prime-test delay was shorter than the subordinate prime-test delay (by approximately 15 minutes), this finding suggests that even when the meaning of an ambiguous word is encountered that is already (on average) preferred by participants, it is still possible to boost its availability. As a result of the different prime-test delays, the size of the dominant and subordinate meaning priming effects cannot be directly compared, although Rodd et al. (2013) provide evidence that larger priming effects can be seen for the more highly subordinate meanings, indicating that the initial dominance of the primed meaning may indeed moderate the magnitude of priming.

These subordinate and dominant priming findings are consistent with our current view of lexical-semantic representations (Rodd et al., 2013, 2016), which suggests that the mechanism for updating word-meaning representations involves changes to connection strengths among units in a connectionist network (Rodd et al., 2004). According to this view, each individual encounter with a word-meaning strengthens the relevant connections in proportion to the overall frequency with which each meaning is encountered. This theoretical view would therefore predict that an encounter with either the subordinate or the dominant meaning would alter the connection strengths related to the representation of the word’s subordinate or dominant meaning, respectively, increasing the availability of the relevant meaning representation so that when the word is later encountered in isolation, there is a relatively greater bias towards interpreting the word with this same meaning. In other words, Experiment 1 shows that lexical-semantic representations are sensitive to a single meaning encounter regardless of the initial availability of the meaning itself (i.e., whether it is the dominant or subordinate meaning). This is consistent with our view that lexical-semantic representations are dynamic even in adults, such that they flexibly adapt to reflect the up-to-date likelihood of occurrence to maintain efficient processing of ambiguous words.

### Cumulative Effects of Multiple Encounters

Experiments 2 and 3 go beyond previous findings in showing that repeated word-meaning encounters within a relatively short period of time (e.g., 20–30 minutes) can lead to cumulative effects in updating the representations of word-meanings similar to those shown in the literature (Rodd et al., 2016) with longer-term (e.g., days/months/years) cumulative effects from experience with ambiguous words. Both Experiments 2 and 3 showed that three spaced encounters of the same subordinate word-meaning biased the later interpretation of that word (in isolation) towards that subordinate meaning over a single encounter. The impact of three spaced repetitions was not threefold the magnitude of one repetition: this is consistent with an asymptotic nature of repetition effects found in the repetition priming field, such as with a lexical-decision task (Logan, 1990). This finding is consistent with previous accounts of word-meaning priming and the view that the effect of experience is cumulative. In contrast, it rules out an account of word-meaning priming in which only the most recent encounter is critical in determining the accessibility of word meanings. This latter view predicts that there would be no difference between the one and three spaced conditions, as they both involved the same single sentence encounter with the subordinate meaning as the most recent encounter of the word. However, this was not the case; three spaced subordinate repetitions made participants more likely to retrieve the subordinate meaning at test. Thus it is not only the most recent encounter that affected word interpretation, it is the effect of multiple recent encounters of the same meaning that accumulate to produce an additional influence on later interpretation.

Furthermore, Experiment 1 showed a residual effect of the initial subordinate meaning even after a subsequent encounter with the dominant meaning; there were more subordinate responses when the subordinate prime had preceded the dominant prime than when the dominant prime had been presented alone. Again, if only the most recent encounter were critical, the subordinate plus dominant condition and the dominant only condition would show equal priming, as they both involve the same dominant prime sentence being encountered most recently. As the former condition resulted in more subordinate responses than the latter, we can conclude that the dominant meaning does not completely ‘cancel out’ the earlier subordinate encounter, rather, the effect of the recent dominant encounter in fact *adds* to the effect of the earlier subordinate encounter. Once more, it is the cumulative effect of multiple recent encounters of different meanings that combine to influence interpretation.

In summary, these data provide clear evidence that multiple encounters with ambiguous words can, when spaced throughout the prime phase, have a cumulative effect on how these words are interpreted in the future. We have now shown that for repeated encounters with the *same* meaning (Experiments 2, 3) and for repeated encounters with *different* meanings (Experiment 1), subsequent interpretation is not driven solely by the individual’s most recent encounter with that word. These data can only be explained by assuming that recent experience with word meanings can accumulate across multiple exposures, such that earlier experience with the word-meanings is not fully overwritten by the most recent encounter. This aspect of the data is fully consistent with the mechanism put forward by Rodd et al. (2013) to explain how lexical-semantic representations update. The proposed mechanism involves changes to connection strengths among units in a connectionist network, which would allow transient changes in meaning availability to accumulate slowly across the lifespan based on each individual experience with a word. These changes appear to reflect a build-up of evidence about the relative likelihoods of different word-meanings across a wide range of timescales. In this view, lexical-semantic representations subtly but continually update based on experience with word-meanings, so that these representations adapt dynamically to the listener’s environment. This view is consistent with the finding that rowers show a long-term preference for rowing-related meanings that increased for those rowers with more years of rowing experience (Rodd et al., 2016).

Although the present findings are lab-based, Rodd et al. (2016) revealed two findings indicating the real-world generalisability of updating meaning representations. First, rowers’ long-term experience with specific meanings generalised to non-rowing settings (they were not informed that it was a rowing-related experiment and the experiment was not performed in a rowing environment). Second the radio study shows that the word-meaning priming paradigm was also successful outside of the lab, as participants heard the prime sentences over a radio show, later finished the experiment in their own time and place (i.e., not in a lab setting) and were not aware that the test was in fact linked to the radio prime phase.

Taken together with these earlier findings, the present results suggest that repeated encounters with a word-meaning gradually strengthen the relevant connections in the lexical-semantic network, which can change an individual’s meaning dominance both in the shorter-term (present experiments) and longer-term (Rodd et al., 2016).

### Benefit for Spaced Over Massed Repetitions

Experiments 2 and 3 demonstrated that when three subordinate meaning repetitions were presented in a spaced manner (i.e., with a 5-minute delay between each), this produced significantly more priming than when only one repetition had been presented. Moreover, Experiment 3 demonstrated that these three spaced repetitions also produced significantly more priming than three massed repetitions (i.e., than when each repetition was presented in succession). It seems that when repetitions were massed, they did not bias responses towards the subordinate meaning any more than one repetition (Experiments 1, 3). Unlike the more general effect of repeated exposures discussed above, this specific spacing (over massed) benefit was not predicted by our current mechanism for updating meaning representations (Rodd et al., 2013). For decades, practice and spacing benefits for memory have been studied using a variety of different paradigms (Karpicke & Bauernschmidt, 2011; Madigan, 1969; Melton, 1970), yet there has been little agreement on the mechanism underlying these spacing effects (Delaney, Spirgel, & Toppino, 2012; Gotts, Chow, & Martin, 2012; Pavlik & Anderson, 2005; Raaijmakers, 2003; Shea, Lai, Black, & Park, 2000). Thus the specific mechanism for the spacing advantage here, as in other memory and learning paradigms, is an ongoing area of debate that warrants future investigation. Furthermore, the word association test used here reflects the ultimate outcome of multiple processes involved in word interpretation, including word recognition, meaning access, and word associate retrieval. Consequently we cannot draw a strong conclusion about which process(es) are affected by the spacing of prior exposures to word meanings, and other measures of word-meaning priming might yield different results.

Previous accounts of word-meaning priming do not provide an explanation for why the extra learning from additional repetitions should be impeded when the temporal spacing *between* repetitions is removed. However, there are two logical possibilities for why the additional massed repetitions do not contribute to learning. One possibility is that learning is primarily driven by the first of the massed repetitions, but is absent (or significantly reduced) for subsequent massed presentations. Alternatively, learning may be driven (primarily) by the most recent of massed repetitions and, for some reason, this final encounter reduces the extent to which the listener learns from the previous massed encounters. Knowing which of these possibilities drives the lack of a massed repetition benefit would help to elucidate the mechanism underlying the updating of meaning representations.

One example of a class of model in which listeners benefit primarily from the first of multiple massed encounters is the activation account (Pavlik & Anderson, 2005, 2008). This model suggests that with each encounter of an item, activation strength increases, but this increase decays as a power function of time. The *rate* of decay is greater when activation is higher, such that the benefit from highly active items will decay faster than for less active items. Hence, providing space between repetitions means that activation has time to decrease between each repetition, thus the rate of decay is slow and the benefit of repetitions lasts longer. Without this spacing between repetitions, as in the massed repetition case, there is not enough time for activation to decrease. This higher initial activation therefore means that the rate of decay is relatively fast and the benefit of massed repetitions does not last as long as for spaced repetitions. This notion is similar to that of a refractory period where, post repetition, there is a period during which activation cannot be further increased by (i.e., is unresponsive to) further repetitions (e.g., Hintzman, Block, & Summers, 1973; Welford, 1952).

In contrast, the consolidation account is an example of a class of model in which individuals learn primarily from the most recent of multiple massed encounters (e.g., Landauer, 1969; and specifically relevant to the present consolidation explanation, proposed for motor skill learning, Shea et al., 2000). This view suggests that memory formation is an ongoing consolidation process following the presentation of a stimulus that can result in transfer from short- to long-term memory, which is more resistant to forgetting and interference (e.g., Brashers-Krug, Shadmehr, & Bizzi, 1996). However, if this consolidation process is interrupted, then the long-term memory does not form properly, or indeed at all. Thus even a new encounter with the same stimulus could interrupt consolidation and reduce or prevent learning (Shadmehr & Brashers-Krug, 1997). Applying this to word-meanings, with three massed repetitions, the memory trace for the first repetition would start to consolidate after presentation but this process would be interrupted by the presentation of the *same* word-meaning just seconds later. As the third repetition is the final encounter, this word-meaning would have more uninterrupted time for consolidation, although it is the only repetition out of the three to consolidate fully, making the massed condition similar to the one repetition condition in terms of consolidation. In contrast, spaced repetitions would show a priming benefit in this account because it allows sufficient time between repetitions for the word-meaning to be (partially) consolidated after each encounter.

Finally, in contrast to these two views, which both assume that it is the timing of the events that drives the observed spacing effect, we must consider an alternative view that this effect is instead driven by differences in contextual variation between massed and spaced exposures. This account proposes that spacing benefits can be explained by an encoding variability mechanism (Maddox, 2016). According to Mensink and Raaijmakers (1989) and Raaijmakers (2003), the general context surrounding a stimulus naturally fluctuates over time and this context is encoded with each presentation of a stimulus. As the temporal spacing of repetitions gets longer, the natural context is more likely to vary and that variation between stimulus encodings increases the likelihood/magnitude of learning from that stimulus. Hence, this account would suggest that the spacing benefit arises due to the increase in different encoded contexts for the spaced word-meaning exposures, which would subsequently make the meaning more available. This model is akin to the concept of contextual diversity (Adelman, Brown, & Quesada, 2006; van Heuven, Mandera, Keuleers, & Brysbaert, 2014), which has been shown to affect word processing (lexical decision performance is better explained by contextual diversity across word occurrences than by just the frequency of occurrence). Similarly, the “One Sense per Discourse” principle (e.g., Gale et al., 1992) is based on the finding that an ambiguous word encountered multiple times within a discourse is highly likely to be used in the same meaning across those encounters, and suggests that an interlocutor would treat one subordinate repetition and three subordinate repetitions within the same discourse/paragraph as equivalents because they both provide *one* overall piece of evidence about *one* meaning (as opposed to *multiple* separate/spaced pieces of evidence of that one meaning).

However, we believe that this encoding variability/contextual diversity/“One Sense per Discourse” type of account is less likely to provide an explanation for the current data. Although this account can explain the observed boost for spaced presentations compared to massed presentations, it cannot explain why three massed repetitions did not boost priming compared to one repetition, given that in Experiments 2 and 3 its two additional repetitions were presented in three separate sentences that did not link together into a coherent discourse. Even in the massed condition, these three sentences provided different contextual information and were distinctly presented in separate pieces of discourse (each sentence was followed by the judgment of relatedness of a probe word, and the sentences were unrelated) so this should provide enough contextual variation to see an increase in priming (compared with one repetition) even for the massed condition and even though the overall situational context did not vary a great deal. Yet, the massed condition provided no additional priming compared to one repetition, despite its two *additional and distinct* sentences/discourses of varying contextual information. While contextual variation accounts consider the general surrounding context rather than context within the sentence, it seems unlikely that additional sentential context would not boost priming if context were such an integral factor in priming. This makes the contextual variation account an unlikely explanation for the present findings. Clearly, it seems that there are several possible mechanisms underlying the spacing benefit but, as aforementioned, this requires further research to disentangle.

Importantly, the observed lack of benefit for multiple massed repetitions is likely to be advantageous from a communication point of view, as these instances are not always representative of the broader word usage. For instance, a conversation with a tree surgeon might involve the tree meaning of ‘bark’ multiple times in a short passage/time-frame of perhaps minutes. If meaning preferences updated cumulatively with each of these repetitions, then this conversation alone would have a disproportionately large effect on meaning preferences for ‘bark’ compared with hearing the same number of ‘tree bark’ repetitions over a longer time-frame of perhaps days or weeks. In this case, the overly sensitive change in meaning preferences would be inefficient. In contrast, if additional word-meaning repetitions only alter representations when sufficiently spaced, lexical-semantic representations might still be somewhat sensitive to the listener’s immediate environment but would primarily reflect the listener’s long-term, temporally distributed (spaced) experience with word usage, which are more likely to accurately predict how these words are used in the future. Under this account, exposure to multiple instances of a word used with its low-frequency meaning would produce a smaller biasing effect on its lexical-semantic representation, and therefore this representation would more likely generalise to future encounters.

### Conclusions

Adults’ lexical-semantic representations are updated dynamically in response to ongoing experience to reflect the most likely meaning of words. The present studies investigated the changes that occur as a consequence of exposure to the meanings of an ambiguous word. The results replicate the word-meaning priming effect and go further in showing that multiple subordinate repetitions provided an additional boost to priming compared with one repetition when these encounters were spaced, although this boost was eliminated when multiple repetitions were massed, at least in a word association test. Moreover, one repetition of the dominant meaning reduced, but did not eliminate, the effect of prior subordinate meaning priming. These results indicate that the experience-based changes to lexical-semantic representations are not solely based on the most recent encounter with a word meaning, nor does the effect occur with the same magnitude across repeated encounters. Rather, word-meaning interpretation appears to reflect the accumulation of recent experiences with word-meanings, where the temporal spacing of multiple encounters is key to producing additional learning effects. This seems to provide a balance among the influences of word usage patterns across a range of timescales, such that listeners can dynamically retune and update their lexical-semantic representations in response to recent experience while maintaining their longer-term knowledge of word-meaning dominance.

## Supplementary Material

10.1037/xlm0000507.supp

## Figures and Tables

**Table 1 tbl1:** Ambiguous Word Repetitions in the Six Experimental Conditions in Experiment 1

Task	Number of items encountered
Subordinate prime task	20 homophones—one repetition
	20 homophones—three repetitions
	[20 homophones—unprimed baseline]
Filler task	(Video)
Dominant prime task	10 subordinate one repetition homophones
	10 subordinate three repetitions homophones
	10 subordinate unprimed homophones
Word association test	All 60 homophones
*Note*. Twenty ambiguous words (in square brackets) were not encountered in the subordinate prime phase but were later included in the word association test to act as an unprimed baseline against which to compare any word-meaning priming effects.	

**Table 2 tbl2:** An Example of the Three Sentences and Probe Words for the Ambiguous Word ‘Glasses’ in Experiment 2

Number	Sentence (ambiguous word in bold)	Probe
1	The cupboard stored the mugs and **glasses**	Prefer (unrelated)
2	She poured the champagne into the **glasses**	Fizz (related)
3	The waiter set out the plates, cutlery, and **glasses**	Table (related)

## Footnotes

1 The age range was restricted to a maximum of 40 years, because older adults may be more receptive to priming than younger adults (Laver & Burke, 1993).

2 All experiments in the current paper were approved by the UCL Division of Psychology and Language Sciences Ethics Committee, fMRI/2013/001.

3 There were no unambiguous prime items.

4 The offset of the spoken word within the auditory file and the presentation of the type-in prompt were not synchronised, which meant that analyses of RTs were not possible for the experiments in the present paper.

5 Whilst logistic mixed effects modelling was used to analyse these data, and those in Experiments 2 and 3, it does not provide “interpretable” means, hence Figure 2 here, and Figure 4 and 6 below, show the subject means. For this reason, there may be some slight discrepancies between the results of LMEM analyses, which account for both item- and subject specific effects, and the results implied by the subject means in the figures.

6 Where the slopes were removed, the intercepts were put back into the model.

7 Although a maximal random effects structure does seem to protect against inflated Type I error (Barr et al., 2013), the size of this inflation is still under debate. More recent research has shown that an intercepts only random effects structure does not necessarily inflate Type I error (Matuschek, Kliegl, Vasishth, Baayen, & Bates, 2017), and this model structure is still preferable to the equivalent separate within-subject/item ANOVAs, since LME allows these analyses within a *single* model.

8 We included only the dominant unprimed trials in this analysis, excluding the dominant primed condition, as we were interested in awareness of subordinate meaning encounters only. Each awareness factor was included as a fixed factor in a logistic mixed effects model along with the fixed factor of subordinate priming, which indicated whether an item was unprimed or subordinate primed (i.e., this factor combined one and three repetition items as ‘primed’). The random effects structure was constructed with subjects and items intercepts and slopes for subordinate priming. The interaction between the relevant awareness factor (aim or estimate) and subordinate priming factor was the crucial test, as a significant interaction would indicate that priming varied as a function of the awareness factor. As before, a model comparison approach was used to determine the significance of this interaction, where a model with both fixed effects and their interaction was compared with a model with both fixed effects without the interaction term.

9 The logistic mixed effects models were constructed as in Experiment 1, again with the crucial test being the interaction between the relevant awareness factor (aim or estimate) and subordinate priming factor, as a significant interaction would indicate that priming varied as a function of awareness. Although the models including the subjects and items slopes for subordinate priming failed to converge, the removal of these random effects allowed for convergence, leaving intercepts-only models for both of the following analyses.

10 The logistic mixed effects models were identical to those in Experiment 2 (intercepts-only random effects structures due to convergence failure when slopes for priming were included). As with Experiments 1 and 2, the crucial test was the interaction between the relevant awareness factor (aim or estimate) and subordinate priming factor, as a significant interaction would indicate that priming varied as a function of awareness.

## Appendix

*List of the 60 Experimental Ambiguous Words Used in Experiment 1*

**Table:**

Ambiguous words			
Appendix	Coach	Key	Pupil
Arms	Cold	Lace	Race
Ball	Craft	Landing	Racket
Band	Crane	Letter	Record
Bar	Cricket	Mark	Ring
Bark	Deck	Mould	Spade
Bolt	Drill	Mouse	Spring
Bonnet	Figure	Note	Staff
Break	Gear	Nut	Step
Cabinet	Gum	Organ	Stitch
Cap	Habit	Palm	Straw
Case	Interest	Panel	Strike
Change	Iron	Pipe	Temple
Cheek	Issue	Pride	Trailer
Chest	Jam	Punch	Watch

*List of the 88 Ambiguous Words Used in Experiments 2 and 3*

**Table:**

Ambiguous words			
Appendix	China	Joint	Racket
Ball	Coach	Key	Record
Band	Cold	Knight	Ring
Bar	Craft	Lace	See
Bark	Cricket	Landing	Sign
Bat	Cross	Letter	Sink
Bed	Cup	Mark	Skip
Blew	Deck	Match	Son
Bonnet	Drawer	Mould	Spade
Bow	Fan	Mouse	Speaker
Bowl	Fence	Nail	Spring
Box	Figure	Note	Staff
Break	Flour	Organ	Step
Bulb	Gear	Pair	Stitch
Button	Glasses	Palm	Straw
Cabinet	Gum	Panel	Strike
Calf	Hand	Park	Temple
Cap	Hare	Pen	Toast
Card	Interest	Pipe	Trailer
Case	Iron	Plug	Trunk
Change	Issue	Punch	Watch
Chest	Jam	Pupil	Wave
