# Supplementary material for: Retuning of Lexical-Semantic Representations: Repetition and Spacing Effects in Word-Meaning Priming
Source: J Exp Psychol Learn Mem Cogn. 2017 Dec 28;44(7):1130–50. doi: 10.1037/xlm0000507 (PMC6012009; doi:10.1037/xlm0000507)
Supplement: Supplementary file 1 [file SuppMaterials_RetuningOfLexicalSemanticRepresentations.docx]

Manuscript title: Retuning of lexical-semantic representations: Repetition and spacing effects in word-meaning priming

# Supplementary Materials

Below are the subordinate prime task stimuli (paragraphs and their summaries) from Experiment 1. The ambiguous words are shown in bold. The substitute words of similar meaning are shown in bold italics (for the one repetition condition), which were replaced by the ambiguous words for the three repetition condition version of the paragraphs.

1. Appendix

Hidden in the **appendix**, at the end of the book, were the thoughts and memos of the author. It was clearly one of the first printed copies of the book, kept by the author’s family. The memos are likely to have been added in the ***back*** as an afterthought, perhaps with the aim of one day being transferred from the ***back*** and added to the book as a second edition.

Summary: the author’s family kept his memos

1. Arms

The government is refusing to provide the rebels with **arms**, despite acknowledging that it is in desperate need of help. Critics warn that whilst providing medical support and armour for defence is helpful, the rebels will be unable to succeed without our government giving them ***weapons***. The critics argue that without these ***weapons***, the rebels will not be able to create a great enough impact, meaning that the war will be prolonged.

Summary: government medical support for rebels is insufficient

1. Ball

This year’s charity **ball** has raised an enormous amount of funds compared to the same ***event*** last year. A staggering 450 people turned up to show their support for the charity and to donate funds in a more fun and engaging way. The a-list guests included the Beckhams, Brad Pitt and Angelina Jolie, all known for their charity work. Guests commented that the ***event*** had been particularly well organised.

Summary: A-list celebrities show their support for charity

1. Band

The charity propose to give out a **band** to everyone walking through their doors, to help raise awareness of the amount of work yet to be done, which needs to be funded. It is hoped that this ***symbol*** will act as a constant reminder to people to donate cash to the cause. If more people wear this ***symbol***, more attention should be paid to the charity, increasing awareness.

Summary: charity to increase awareness by giving out free gift

1. Bar

A metal **bar** connected the two wheels at the front of the make-shift toy car and another metal ***pole*** connected the rear two wheels. Jimmy had used an old wooden crate he found in the garage left over from when his family owned a fruit and vegetable stall on the market. A third metal ***pole*** supported the crate underneath so that it could take Jimmy’s weight, also connecting the two sets of wheels.

Summary: Jimmy makes use of old objects to make a toy car

1. Bark

The woodpecker clung onto the **bark** with its feet, hammering incessantly at it. The green and red markings on the bird’s head blurred with the speed of the pecking, almost blending in with the mottled brown of the ***trunk***. Every so often it would stop to check for predators although it did not often encounter them, thanks to it being well camouflaged when it was on the ***trunk***.

Summary: woodpecker’s markings provide adequate protection from predators

1. Bolt

The rumble of thunder amidst the sound of the heavy rain drew closer and closer. A **bolt** sparked across the slate grey sky, followed almost immediately by the clatter of thunder again. Another ***flash*** made a fork of light in the sky, coming dangerously close to a nearby tree. Each ***flash*** of lightening terrified the animals on a nearby farm, making them huddle together under a small shelter.

Summary: the effect of a powerful thunderstorm causes animals to search for shelter together

1. Bonnet

Ladies used to wear a **bonnet**, which covered most of their hair. It turned up at the front, was tied with ribbon under the chin and was often finished with a frill across the top to make them appear more elegant. Such an ***accessory*** almost became a representation of status; the more embroidered, the higher the woman’s status. This embellished ***accessory*** was worn by the writer Emily Bronte, for instance.

Summary: the more decorative a ladies clothes, the higher her status

1. Break

Annie had locked herself out of her own house. The only way she could think of getting in was to **break** a window. If she could ***smash*** a small window, it wouldn’t cost much to replace it, and she was petite, meaning it would be relatively easy for her to climb through the small gap. The problem was that she had no implement with which to ***smash*** it.

Summary: the easiest and most cost effective way of entering a locked house

1. Cabinet

The **cabinet** concluded that a referendum would be unnecessary, since the time it would use might only worsen the financial situation. The ***politicians*** had been in talks for several weeks about a plethora of problems, but had only discussed the idea of a referendum over the last few days. Their decision was not a popular one, since previous ***politicians*** held many referenda, which had proven popular with the public.

Summary: alteration in government’s tactics prove unpopular

1. Cap

Students have proposed a lower **cap** on tuition fees as the government pass the increase in university costs. A poll revealed that students think a tuition fee ***limit*** of approximately £5,000 would be a more acceptable and realistic ***limit*** than the current sum of around £9,000. They understand that the government will not bring the costs down to the previous rate, therefore they are fighting for a compromise.

Summary: students seek a reduction in the current cost of university fees

1. Case

Police have now confirmed the **case** to be closed after the hearing, which spanned several months, resulted in a twenty-year-old man being charged for the offence. Investigators had been unable to comment in detail on the ***matter*** until the length of the sentence had been confirmed. This ***matter*** has been one of the most publicised this year, sparking many debates in the House of Commons due to its highly controversial nature.

Summary: man finally charged after lengthy court hearings

1. Change

The man checked his **change** several times. Being an honest customer, he walked all the way into town, to the supermarket in which he had been a regular customer for years. He told the cashier that she had given him too much ***money***. The manager of the supermarket allowed the customer to keep the ***money*** as a token of gratitude for his honesty. They valued good customer service.

Summary: honest customer rewarded for returning overpayment

1. Cheek

“Honestly, Sophie, it’s just the **cheek** of it!” exclaimed the teacher. She couldn’t believe that a student had spoken to her in that manner; she was constantly harping on about respect for one’s elders and teachers. “I’m utterly shocked at your ***rudeness*** – I don’t know what to say”, the teacher continued. “I cannot believe any student would behave in that way towards me; such ***rudeness***”, the teacher added repetitively.

Summary: teacher reprimands student for bad behaviour

1. Chest

The professor kept his unpublished manuscripts in a large wooden **chest** underneath his desk, so that anyone looking through his files wouldn’t come across them. Also in the ***box***, he guarded his theories, which he planned to reveal upon gaining sufficient evidence for them. The only other person aware of the ***box*** was the cleaner, but he had been a friend of the professor for years, so the professor felt that he was trustworthy.

Summary: professor trusts cleaner with details of unpublished work

1. Coach

Team GBs head **coach** for cycling emphasised the importance of the acoustics in the velodrome – the noise of the British crowd really spurred the cyclists on and helped them to achieve their medals. He commented on how proud he was to be the ***instructor*** in the home Olympic Games of 2012 and that the role of the ***instructor*** was a stressful but equally rewarding role, an opportunity not to be missed.

Summary: GB cycling chief recognises design of velodrome as crucial to team’s success

1. Cold

It was that time of year in the surgery where every patient waiting to see a doctor seemed to have a **cold**. Even the receptionist had some sort of ***virus***, someone whose immune system you would think would be near invincible. Even many of the doctors had been off work complaining of the symptoms of the ***virus***, leaving the few remaining doctors with even more patients than usual.

Summary: seasonal flu takes hold, affecting doctors as well as patients

1. Craft

During World War II, a **craft** called the Strathaird was used to transport troops from England to Algeria. The journey took three weeks. Before the war, the ***ship*** was a luxury cruise liner, carrying ‘first-class’ and ‘tourist-class’ passengers. The ***ship*** was built with three funnels along its length, even though the first and third funnels were dummies to make it appear more powerful. The two dummy funnels were removed after the war.

Summary: luxury cruise liner transformed to war vessel

1. Crane

Origami is the traditional Japanese art of paper folding, which remains popular and has even influenced the fashion world. You can make anything from a bird, such as a **crane**, to a cobra, although the ***bird*** is more suitable for beginners, as it is relatively quick and simple to create. It is an important animal for the Japanese, who sometimes refer to ***it*** as ‘the bird of happiness’.

Summary: making Japanese origami can be quick and simple with some designs

1. Cricket

The noise of the **cricket** can be incredibly loud, particularly so for an insect. Tourists visiting the ancient Minoan settlement in Knossos, Crete, cannot ignore the sounds of the ***insect***, which echo throughout the ruins of the ancient civilisation. They predominantly stay in the vegetation on the largely barren land. The ***insect’s*** noise is created when they rub their wings together, although it is only the males of the species that produce sound.

Summary: on the island of Crete, bugs hidden in the vegetation are very noisy on a hot day

1. Deck

The magician asked the volunteer to pick a card from the **deck** but not to reveal it to anyone. The volunteer looked at the card, memorised it, and returned it to the ***pack***. The magician spent a few seconds shuffling the cards before continuing with the trick. He threw the ***pack*** in the air, collected them from the ground and then pulled one card out - the card that the volunteer had picked.

Summary: magician amazes volunteer with card trick

1. Drill

For each trainee that failed the exercises, everyone in the team had to do an extra **drill**. Many of the trainees had never worked so hard in their lives, and so they struggled to complete the extra ***exercises***. One lazy trainee, who failed on several occasions, proved to be rather unpopular in the team, not surprisingly. Without him, the team would not have had to complete seven extra ***exercises***.

Summary: lazy trainee makes extra work for others

1. Figure

The current **figure** for the world’s population stands at approximately 7 billion, and it is thought that this ***number*** will increase to around 10 billion by 2050. However, the United Nations is concerned that the population might exceed the estimated ***number*** due to increased life expectancy. If this is true, it will result in a significant increase in demand for land and resources, such as water and crops that will not be sustainable.

Summary: increasing life expectancy causes concern for future resources

1. Gear

After donning her tatty swimming **gear**, Lucy reluctantly walked out of the changing rooms and into the indoor pool area. Her peers all had the proper ***costume***, but Lucy’s family could no longer afford to provide her with the ***costume*** with the school emblem embroidered onto it. Lucy wouldn’t have minded too much normally, but because she was so poor at swimming it meant that all the attention was on her.

Summary: Lucy at a disadvantage in swimming causes her to feel unhappy

1. Gum

The dentist’s assistant had thoroughly sterilised the equipment but the patient still hated the idea of it all having been used on lots of other people before. The dentist picked the sharpest looking tool and prodded the patient’s upper **gum**. He then moved onto testing the lower ***mouth***. He was checking for disease and wanted to do it thoroughly, since it can be a serious health problem regarding the ***mouth***.

Summary: unconvinced patient reluctantly allows dentist to examine him

1. Habit

A **habit** is the type of clothing worn by monks and nuns. It is made up of a tunic and a hood for monks and veil for nuns, and is typically brown or black, respectively. The ***uniform***, typical of Roman Catholic monks and nuns, serves to make the person identifiable as of the religion. However, some more modern nuns wear only the veil, as opposed to the entire ***outfit***, as it is more practical.

Summary: traditional religious attire proves impractical

1. Interest

**Interest** is charged by lenders against funds leant to both businesses and consumers. The bank of England base rate is abnormally low at present as a result of the economic climate. Many businesses are only surviving because of these low ***monetary*** rates. The government are supporting some banks, which are effectively owned by the tax payer, by lending them funds at low rates. These rates are passed on, plus the bank’s ***margin***.

Summary: economic climate forces government to support banks

1. Iron

A simple school science experiment involves **iron** filings. You put the ***filings*** onto paper and put two magnets with south ends pointing towards each other. The filings will be repelled, forming lines pointing in the opposite direction, away, from the magnets. The pattern of the ***filings*** demonstrates the magnetic fields of the magnets and is easier for young school children to understand than an explanation of the effect alone.

Summary: practical science experiment simplifies explanation of magnetic fields

1. Issue

Vogue, an American fashion magazine, was first published in 1892 with one **issue** per week. It was later published in Britain in 1916 as a bi-weekly magazine, after being taken over by Condé Nast in 1909. The first British ***publication*** features an illustration of a puppet show drawn by Helen Thurlow. This ***copy*** of Vogue was the start of the magazine’s international success, turning into a monthly style and fashion beacon.

Summary: the story of Vogue from its humble beginnings

1. Jam

There has been a **jam** on the M11 for several hours now following a lorry crash between the junctions for Bishop’s Stortford and Harlow. Motorists are advised to take other routes, although, as a result of the incident, there is also a ***queue*** on the A120 due to volume of vehicles avoiding the M11. There’s also another ***queue*** on the M25, clockwise, but we are awaiting further details on where exactly it is.

Summary: motorway accident causes local A-road congestion

1. Key

The young musician had spent weeks writing his piece of music. He had studied other classic pieces that had the same **key** that he wanted to use for his composition. His music teacher disagreed with the ***tone*** he used, but the musician was determined to stay true to what he believed would work and continued using the same ***tone***. He had been heavily influenced by the classic pieces he had studied.

Summary: stubborn musician disagrees with teacher over style of work

1. Lace

As she fell, Alice looked behind her to see what she had tripped on, but there was nothing on the path. She hit the ground, grazing her hands and one of her knees in the process, but rapidly picked herself up. She noticed that it was the **lace** on her new brogues that she had tripped on. She tied ***it*** up again, checked the ***one*** on the other shoes, and carried on.

Summary: Alice trips over because of her shoes

1. Landing

There was very little space on the **landing** on the first floor. The cluttered shelves were deep as well as tall, leaving only about a one metre length of ***space***. The shelves were crammed with souvenirs from holidays and exotic places, each covered in a thick layer of dust. On the ***floor*** above it was similar, but the shelves were littered with old books and folders instead of souvenirs.

Summary: house clutter leaves little space for anything else

1. Letter

The little girl sounded the word out one **letter** at a time. She struggled on the last ***one*** but managed to read the word eventually. She was fairly confident, and had the right to be so, since she was above average in her class with her reading skills. She had practised the alphabet from a very young age and was keen to improve when there was a ***character*** she couldn’t pronounce well.

Summary: conscientious student persistently attempts to improve her standard

1. Mark

I was extremely disappointed with my **mark** so I asked my new tutor to read my essay for me – I wasn’t convinced that my previous tutor had even read it properly, therefore giving an inaccurate ***grade***. I thought that if I could just get one more ***point***, it would be good. That could be the difference between two grades at the end of the year, following exams.

Summary: keen student presses tutor on important essay

1. Mould

The best thing about this clay-like substance is that you can easily **mould** it into whatever shape you want. Kids love to make snowmen out of it, especially at this time of year. You just ***make*** three different sized spheres and put them one on top of the other, starting with the largest. If it dries out, which isn’t often, you can add a little water to make it easier to ***shape***.

Summary: medium used for making pretend snowmen for children

1. Mouse

The Apple iMac, 21.5 or 27 inch screen, comes with a wireless state-of-the-art **mouse**. This ***device*** is ergonomically designed, so that it fits into the hand comfortably. The user experience is different from what many people are used to with Windows computers. In addition, different finger movements on the ***device*** allow users to zoom in and out, as well as flick between different pages and documents on the computer.

Summary: new technology impresses Apple customers

1. Note

Since his voice had broken, he was no longer able to reach the **note** that had made him so famous as a singer. The ***pitch*** that even some girls couldn’t reach, or if they could, he could usually do it better than they were able to. This demonstrated his magnificent musical ability. Despite no longer being able to reach a particularly high ***pitch***, he remains a very successful artist.

Summary: despite a difference in voice, singer remains at the top of his game

1. Nut

The machine has failed due to a very simple fault; the **nut** that held together the two central pieces of the mechanism had fallen off. The engineer screwed the ***part*** on and the machine worked once more. It was an unexpectedly cheap problem to fix. The worker knew that if the ***part*** were to fall off again in the future it was something that he could fix himself.

Summary: machine failure in factory easy to repair

1. Organ

The bride, who felt incredibly nervous, slowly put one foot in front of the other, making her way gracefully down the aisle. The **organ** music filled the church with a glorious atmosphere, making the guests feel more excited still, whilst awaiting the bride. The player played the ***wedding*** piece flawlessly, which would be expected; she had played the ***instrument*** since she was a teenager, so had had plenty of practice.

Summary: the experienced musician entertained the bride, groom and guests

1. Palm

The beach stretched for miles along the coastline, with its golden sand and tall **palms** that rivalled that of the most beautiful beaches in the Caribbean. The ***trees*** casted star-like shadows here and there, where the blazing hot sun was beating down on them. Under these ***trees*** were there few holiday makers who could bear the midday heat. The natives were sensible enough to stay inside during this time of day.

Summary: in the Caribbean, the natives know not to sit in the hot sun, unlike tourists

1. Panel

The left **panel** of the car had a scratch along it where the football hooligans had let out their anger. There was also a dent on the other ***side*** where someone had hit it with a brick or something similar. The front and rear ***sections*** of the car were not damaged. The police are now using CCTV evidence to identify the hooligans in order to track them down and prosecute them.

Summary: modern technology used by police to identify hooligans attacking car

1. Pipe

His grandad pondered for several moments, raising his **pipe** to his face and taking in a large puff before putting ***it*** down. He continued to ponder for a while, keeping his grandson in suspense. The ***smoking*** seemed to help him think, and made him feel wiser, or so his grandson thought. The old man sat up in his chair and finally answered in his deep, uneven voice.

Summary: grandson waits in anticipation of pearls of wisdom from his grandfather

1. Pride

The older lion, the leader of the **pride,** appears to be protecting his fellow group members, particularly his two cubs, who are only one month old. The females are also very wary and are staying near the cubs. The ***group*** isn’t large, but it is strong; it comprises of some of the best lions in the area. This is the main reason for the survival of their whole ***group*** in the wild.

Summary: male lion protects cubs in continual fight for survival

1. Punch

As typical teenagers, the girls made the most of the copious amounts of free **punch** served at the pub. The ***drink*** didn’t contain a large amount of alcohol, but the thought of drinking so many glasses of the ***drink*** made the girls act more intoxicated than they were. Their behaviour was somewhat outrageous, although many of the guests decided to ignore them, aware of the fact that they were new to partying.

Summary: guests make the most of a free night out

1. Pupil

Lizzie was the best **pupil** in the class. Many of the other ***students*** were jealous and so they refused to make any effort to get along with her. But Lizzie wasn’t bothered by it. She had friends outside of school, in the village in which she lived. Lizzie knew that being the best ***student*** would be worth it in the long run, especially having such a proficient teacher.

Summary: unpopular with classmates, Lizzie continued to thrive under excellent teacher

1. Race

There has been controversy in the Big Brother house again with a new **race** row between two contestants of a different ***ethnicity***. The producers of the reality television series intervened, via Big Brother, in an argument between the contestants when problems regarding ***ethnicity*** were brought up. The trouble is being investigated. The contestants concerned have been warned that their actions could jeopardise their place in the Big Brother house.

Summary: investigation into racist comments made in Big Brother house

1. Racket

The girl was unable to concentrate with the **racket** going on outside the exam hall; she felt like she couldn’t even hear her own thoughts. It was the invigilator’s responsibility to keep the ***noise*** down, but he was preoccupied with observing a child whom he thought to be cheating. The ***noise*** went on for a while, probably from students that had already finished their exams, being selfish.

Summary: invigilator preoccupied with cheating student causes other students to struggle

1. Record

Today is now officially the hottest day on **record** in August since the first ***archive*** was made in 1914. This is surprising given the unseasonably miserable weather we have been experiencing this summer. There are many ***documents*** on the weather dating to as long ago as the 1600s, although these are not usually referred to since they were not official and, as a result, might be unreliable sources of information.

Summary: hottest day for nearly 100 years is surprise after unseasonably miserable weather

1. Ring

“I’ll give you a **ring** every day”, her husband reassured her. “No, it’s alright, just give me a ***call*** when you get there so I know you’re safe, otherwise I’ll worry”, she replied. She knew she would miss her husband dearly, so thought it would be easier not to talk to him every day. She became desperate to ***call*** him after a few days without contact, but kept to her word.

Summary: a woman sticks to her promise not to speak to her husband while he is away

1. Spade

The gambler slowly placed down his final card with a smug look on his face, savouring the moment. His opponent, Nick, looked at the gambler’s **spade** and then looked at the ***card*** in his own hand. Nick had the highest value. The gambler started to collect his winnings as Nick placed down his card. The gambler’s smug expression soon vanished as he saw what ***card*** his opponent had.

Summary: gambler forced to retreat after fellow player delivers better hand

1. Spring

The slinky, a nostalgic toy for many people over the course of several decades, has a wonderfully simple design. The **spring** cascades from one stair down to the next with ease, and little effort from the player. It was once the plain silver of a mechanical ***coil***, but it now usually multi-coloured. The simple ***coil*** design has been sold to millions of people across the world over the decades.

Summary: nostalgic children’s toy sells despite modification

1. Staff

As the story goes, Moses, an important prophet in many religions, often had a **staff** by his side during important times. According to the Bible, he used this ***rod*** to perform miracles, including turning it into a snake and then into a ***rod*** again, and getting water from a rock. It is also associated with the start of the plague on the Egyptian people, caused by Moses.

Summary: Moses used miracles for several important demonstrations

1. Step

“You need to take your physiotherapy one **step** at a time, Mr Smith, quite literally”, the doctor advised. “If one ***stride*** tires you out, then you must stop there and get yourself used to that one ***stride*** before you push yourself further. You should see a gradual improvement over the weeks to come, as well as in increase in stamina and muscle strength. But you must be patient”.

Summary: physiotherapist warns patient not to expect too much too soon

1. Stitch

The runner had to push through the **stitch**, which came a lot earlier than she was used to. Unfortunately, she did not beat her personal best for the distance. She put this down to the early ***cramp***, which affected her plan for the run. Other athletes, however, experienced a ***cramp*** at a better time in the occasion than they were used to. This also explains her position being lower than usual.

Summary: muscle fatigue affects athlete’s performance

1. Straw

On the **straw** lay the foal, just days old. She was brown, with a light brown mane and tail, like her mother. She chewed at the ***hay*** for a while, before unsteadily standing up and making her way across the stable to feed. The mother was also feeding on the ***hay*** at the time and seemed annoyed at her foal attempting to feed at that particular moment.

Summary: a newborn foal and her mother feed together

1. Strike

The **strike** rivalled the May 1968 ***protest*** in France, when the French economy was almost brought to a standstill. The infamous May 1968 ***protest*** started in Paris with students and snowballed, eventually involving nearly one quarter of the country’s population some two weeks later. It almost devastated the then-current government. Governments since then have learned from the mistakes made, although this does not mean that workers have resisted the temptation to demonstrate their feelings.

Summary: French people show dissatisfaction with government’s economy

1. Temple

The physical symptoms of stress include muscle aches, sweating, alterations in bowel regularity, and headaches, although many more symptoms can be present and the prevalence of symptoms depends on the individual. The headaches tend to be across the forehead and **temple** region, hence the stereotype of a stressed person rubbing the head – the ***side*** is a pressure area and it is satisfying to massage the ***sides***, relieving some tension.

Summary: stress manifests itself in several physical ways

1. Trailer

The **trailer** for the new James Bond film, starring Daniel Craig, was released yesterday, after a long wait from fans due to problems with the film studio. As is clear from the ***clip***, there will be lots of action and drama, sure to make audiences sit on the edges of their seats. The film will not be released until November; another long wait after the release of the ***clip***.

Summary: snippets of new Bond film tease audiences

1. Watch

The boys had played out in the street in front of their houses since they were toddlers and were well-practiced at games such as hide and seek, although they were running out of places in which to hide. One would often keep **watch** from a tree for interfering girls riding around on their bikes. Dominic was the best at keeping ***guard***, so keeping ***guard*** was usually his job.

Summary: Dominic was the most useful in ensuring that the game ran smoothly

***Table 5.*** The 60 sentences and probe words used in the dominant prime task (semantic relatedness task) from Experiment 1. The ambiguous words are shown in bold.

| **Dominant Sentence** | **Probe Word** | **Related** |
| --- | --- | --- |
| The doctor advised removing the patient’s **appendix** | hospital | Y |
| She waved her **arms** frantically to stop the car | road | Y |
| The **ball** rolled slowly into the goal | sport | Y |
| The **band** is doing a concert tomorrow | music | Y |
| Happy hour in the **bar** is from 7 until 8 | cheap | Y |
| The cat jumped at the sound of the **bark** | fear | Y |
| He screwed the **bolt** on tightly | fix | Y |
| There was smoke coming out of the car **bonnet** | engine | Y |
| The new employee worked through her **break** | lunch | Y |
| The cherry wood **cabinet** looked magnificent | furniture | Y |
| He wore a **cap** to shield his face from the sun | bright | Y |
| He hid the confidential documents in his **case** | secret | Y |
| The department made a **change** to the deadline dates | essay | Y |
| He kissed her on the **cheek** as a goodbye | partner | Y |
| The bodybuilder worked on his **chest** muscles | strong | Y |
| Travel by **coach** from Oxford to London is easy | journey | Y |
| The house was **cold** without the central heating | temperature | Y |
| The worker was an expert at his **craft** | tools | Y |
| The builders had to get a **crane** to lift the materials | construction | Y |
| The playing field is mainly used for **cricket** | grass | Y |
| There were chairs and a table on the **deck** outside | garden | Y |
| A **drill** is needed for putting shelves up properly | screw | Y |
| The woman admired her **figure** in the dress | outfit | Y |
| The car was left in **gear** | clutch | Y |
| He spent ages trying to remove the **gum** from his shoe | sole | Y |
| They had a **habit** of lying to their friends | truth | Y |
| He demonstrated his **interest** by attending every lecture | university | Y |
| The housewife got distracted and left the **iron** on | appliance | Y |
| The **issue** had been sorted out by the estate agents | house | Y |
| The schoolboy had a **jam** sandwich for lunch | food | Y |
| The old **key** got stuck in the lock | vase | N |
| The **lace** veil was kept in good condition | glass | N |
| The **landing** was smoother than the flight | paper | N |
| The postman delivered the **letter** on time | ocean | N |
| There was a **mark** on the paintwork | apple | N |
| **Mould** was beginning to grow in the bathroom | time | N |
| The woman screamed when the **mouse** ran near her | style | N |
| The teacher made a **note** to speak to the parents | luck | N |
| She had a severe **nut** allergy | prison | N |
| The man went to register as an **organ** donor | canvas | N |
| She clutched the piece of paper in her **palm** | computer | N |
| The **panel** of judges was not impressed | screen | N |
| The **pipe** for the gas was illegal | early | N |
| The man felt a sense of **pride** after the meeting | mirror | N |
| He had received a **punch** on the jaw | slipper | N |
| His eyes were so dark you couldn’t distinguish the **pupil** | flat | N |
| Her daughters won the three-legged **race** | acrylic | N |
| He threw the **racket** in frustration after losing | pink | N |
| The **record** player was covered in dust | life | N |
| The jeweller sold his most expensive **ring** | air | N |
| The **spade** was too small to dig the hole | rule | N |
| **Spring** was her favourite season | cell | N |
| The **staff** were unhappy with the boss | attach | N |
| She stumbled on the **step** up to her front door | report | N |
| A **stitch** had come loose in the jumper | danger | N |
| The **straw** in the juice carton was split | shake | N |
| He resisted the urge to **strike** back | plate | N |
| The **temple** ruins were covered in moss | high | N |
| The **trailer** had come away from the van | attend | N |
| He kept checking his **watch** while he was waiting | clear | N |

***Table 6.*** Subordinate prime task sentences and probe words from Experiments 2 and 3 (semantic relatedness task). There are three different sentences per ambiguous word and either one or two of the sentences per word have an unrelated probe word paired with it. Ambiguous words are shown in bold.

| **Word** | **Sentence** | **Probe** | **Related** |
| --- | --- | --- | --- |
| Appendix 1 | The author put his memos in the **appendix** of the book | writer | Y |
| Appendix 2 | The student annotated throughout the book, including in the **appendix** | pages | Y |
| Appendix 3 | Additional information to the book was included as an **appendix** | abrupt | N |
| Ball 1 | There were many professional dancers at the **ball** this year | dirt | N |
| Ball 2 | The charity **ball** raised an enormous amount of money | donations | Y |
| Ball 3 | An unprecedented number of guests attended the summer **ball** | subdued | N |
| Band 1 | Everyone in the group wore a **band** | sky | N |
| Band 2 | The charity encouraged people to wear a **band** to raise awareness | dry | N |
| Band 3 | Members had to wear a **band** to identify themselves | group | Y |
| Bar 1 | Access was prevented with a long wooden **bar** | slim | N |
| Bar 2 | A metal **bar** connected the two wheels | vehicle | Y |
| Bar 3 | The gate was made of a wooden bar connected to four planks | barrier | Y |
| Bark 1 | The branches and the **bark** had been damaged by the storm | closed | N |
| Bark 2 | The woodpecker clung onto the **bark** with its feet | bird | Y |
| Bark 3 | The outer layer of the **bark** was starting to peel off | wood | Y |
| Bat 1 | The fruit **bat** is a flying mammal | wings | Y |
| Bat 2 | The wingspan of the largest **bat** is 180cm | fly | Y |
| Bat 3 | There are many species of **bat** | curtain | N |
| Bed 1 | There were weeds growing in the **bed** | garden | Y |
| Bed 2 | There were many varieties of plant in the **bed** | leaves | Y |
| Bed 3 | The plants in the **bed** were beautiful colours | wrong | N |
| Blew 1 | It was so windy the tent nearly **blew** away | camping | Y |
| Blew 2 | The birthday girl **blew** out the candles | cake | Y |
| Blew 3 | A strong wind **blew** across the field | improve | N |
| Bonnet 1 | Petals decorated the brim of the girl’s **bonnet** | clothes | Y |
| Bonnet 2 | The lady tied the **bonnet** under her chin | exotic | N |
| Bonnet 3 | The baby was dressed in a pink dress and **bonnet** | girl | Y |
| Bow 1 | The girl tied a **bow** around her ponytail | daily | N |
| Bow 2 | The bag had been decorated with a **bow** | ribbon | Y |
| Bow 3 | The present was wrapped with a **bow** | perform | N |
| Bowl 1 | The sportsmen’s **bowl** won the game | measure | N |
| Bowl 2 | It was John’s turn to **bowl** | game | Y |
| Bowl 3 | The last player didn’t have time to **bowl** | soap | N |
| Box 1 | The fighter had to **box** better than he had before | seat | N |
| Box 2 | As part of the exercise class, the students had to **box** | bait | N |
| Box 3 | The champion learnt how to **box** at a young age | child | Y |
| Break 1 | The vase was wrapped so that it wouldn’t **break** | protected | Y |
| Break 2 | Annie had to try and **break** the window | pane | Y |
| Break 3 | The boy was scared that the ornament would **break** | boat | N |
| Bulb 1 | The wire connecting the **bulb** was broken | wise | N |
| Bulb 2 | The filament in the **bulb** had broken | light | Y |
| Bulb 3 | The switch worked but the **bulb** had gone | flap | N |
| Button 1 | Although he was told not to, Fred pushed the **button** | disobey | Y |
| Button 2 | She pressed the remote **button** harder | television | Y |
| Button 3 | The light on the computer **button** flashed | grateful | N |
| Cabinet 1 | The ministry advised the **cabinet** on policy alterations | politicians | Y |
| Cabinet 2 | The meeting of the **cabinet** continued for hours | berry | N |
| Cabinet 3 | The results of the referendum confirmed the opinion of the **cabinet** | holiday | N |
| Calf 1 | The muscle in his leg had weakened, particularly the **calf** | injury | Y |
| Calf 2 | The runner had hurt her **calf** muscle | shiver | N |
| Calf 3 | The patient was also suffering from severe cramps in his **calf** | untidy | N |
| Cap 1 | The purchasers were subject to a spending **cap** | yellow | N |
| Cap 2 | The government have agreed to a **cap** on benefits | money | Y |
| Cap 3 | Students have proposed a lower **cap** on tuition fees | university | Y |
| Card 1 | She didn’t have a Christmas **card** to give him | action | N |
| Card 2 | His Grandad had forgotten to send him a birthday **card** | grandchild | Y |
| Card 3 | Betty still sent a **card** to everyone in her village | day | N |
| Case 1 | The crime was suspected to be a **case** of mistaken identity | division | N |
| Case 2 | Police have now confirmed that the **case** is closed | caption | N |
| Case 3 | Lawyers had fought over the **case** for months | argument | Y |
| Change 1 | The cashier had given the customer the wrong **change** | supermarket | Y |
| Change 2 | The machine had run out of **change** | avoid | N |
| Change 3 | The man checked his **change** several times | edge | N |
| Chest 1 | The large wooden **chest** was covered in dust | dirty | Y |
| Chest 2 | The carpenter had only taken a day to make the **chest** | wood | Y |
| Chest 3 | Tom put his keepsakes in a small **chest** under his wardrobe | start | N |
| China 1 | She bought a new set of **china** for the tea party | truck | N |
| China 2 | The host had a large cupboard full of **china** | tea | Y |
| China 3 | One of the guests had smashed a piece of **china** | test | N |
| Coach 1 | Lee was the most respected **coach** in the business | add | N |
| Coach 2 | Team GB has announced their new head **coach** | burn | N |
| Coach 3 | The school had hired a new sports **coach** | education | Y |
| Cold 1 | All the employees had caught the same **cold** that week | value | N |
| Cold 2 | She had felt unwell even before she had the **cold** | engine | N |
| Cold 3 | Alice stocked up on tissues because she had a **cold** | nose | Y |
| Craft 1 | There were three funnels along the **craft** | colour | N |
| Craft 2 | The touchdown space for the **craft** had been cleared | curl | N |
| Craft 3 | The cruise liner had been an old war **craft** | travel | Y |
| Cricket 1 | Only the male **cricket** can produce a sound | noise | Y |
| Cricket 2 | The children searched the garden for a **cricket** | grass | Y |
| Cricket 3 | The wings of the **cricket** make a distinctive noise | dropped | N |
| Cross 1 | His mum was extremely **cross** with him | weather | N |
| Cross 2 | He felt really **cross** after the discussion | argument | Y |
| Cross 3 | Jack knew that his girlfriend would be **cross** | boyfriend | Y |
| Cup 1 | The plaque on the **cup** was engraved | win | Y |
| Cup 2 | The winners held the victory **cup** | scissors | N |
| Cup 3 | The photographer wanted a photo of the champion with the **cup** | lunch | N |
| Deck 1 | The magician asked the volunteer to pick from the **deck** | trick | Y |
| Deck 2 | The blackjack players had their eyes on the **deck** | maid | N |
| Deck 3 | The poker dealer picked up **deck** and shuffled it | gamble | Y |
| Drawer 1 | She kept her gloves and scarves in the **drawer** | hat | Y |
| Drawer 2 | Emily hid the book in the **drawer** | acid | N |
| Drawer 3 | Under the desk, the **drawer** was full | entertain | N |
| Fan 1 | Some celebrities don’t interact with members of their **fan** base | chief | N |
| Fan 2 | The email update was always written by a different **fan** | news | Y |
| Fan 3 | The actress had been stalked by a **fan** | follow | Y |
| Fence 1 | He wanted to learn how to **fence** | sport | Y |
| Fence 2 | The sports society aims to teach people how to **fence** | learn | Y |
| Fence 3 | Using the new sword, he tried to **fence** | art | N |
| Figure 1 | All of the bankers knew what the **figure** would be | attract | N |
| Figure 2 | The accountant had a **figure** in mind | finance | Y |
| Figure 3 | According to politicians, there might be an increase in the total **figure** | government | Y |
| Flour 1 | The bread was made using wholemeal **flour** | healthy | Y |
| Flour 2 | Jane’s food shopping list included eggs, milk and **flour** | elegant | N |
| Flour 3 | The baker used a new type of **flour** | bread | Y |
| Gear 1 | The new employees were told to put on the work **gear** | instruction | Y |
| Gear 2 | Lucy had turned up in her work **gear** | cactus | N |
| Gear 3 | His peers had all the proper **gear** for the contest | competition | Y |
| Glasses 1 | She poured the champagne into the **glasses** | fizz | Y |
| Glasses 2 | The cupboard stored the mugs and **glasses** | prefer | N |
| Glasses 3 | The waiter set out the plates, cutlery and **glasses** | table | Y |
| Gum 1 | The boy could feel his new tooth coming through his **gum** | fade | N |
| Gum 2 | The dog chewed the stick, which cut his **gum** | teeth | Y |
| Gum 3 | The dentist prodded the patient’s **gum** | connected | N |
| Hand 1 | The clock had a broken **hand**, so it didn’t give the time | table | N |
| Hand 2 | The grandfather clock was missing a **hand** | time | Y |
| Hand 3 | The new clockmaker learnt how to attach the **hand** | rotten | N |
| Hare 1 | The fox tried to chase the **hare** | prey | Y |
| Hare 2 | The rabbit is from the same family as the **hare** | picture | N |
| Hare 3 | A wildlife documentary showed the **hare** in its natural habitat | television | Y |
| Interest 1 | The bank charges more **interest** than others | face | N |
| Interest 2 | The lenders charged their clients a lot of **interest** | fee | Y |
| Interest 3 | Many businesses are only surviving because of low **interest** rates | profit | Y |
| Iron 1 | A simple school science experiment involves **iron** filings | students | Y |
| Iron 2 | The engineer thought that **iron** would be a suitable material | small | N |
| Iron 3 | The melting point of **iron** is over 1,500 degrees | chemistry | Y |
| Issue 1 | Alex had edited the most recent **issue** of the publication | left | N |
| Issue 2 | The publishers re-released the oldest **issue** | magazine | Y |
| Issue 3 | Vogue was first published in 1892 with one **issue** per week | fashion | Y |
| Jam 1 | The roadworks caused a **jam** all through the town | construction | Y |
| Jam 2 | All the cars were stuck in a **jam** | malice | N |
| Jam 3 | Shutting the roads for the charity run caused a **jam** | cable | N |
| Joint 1 | The police searched the suspected drug dealer and found a **joint** | duck | N |
| Joint 2 | The teenager smoked the **joint** | illegal | Y |
| Joint 3 | The drug addict only had one **joint** left | credit | N |
| Key 1 | The group wrote the song in a major **key** | sack | N |
| Key 2 | The musician had altered the song’s **key** several times | behave | N |
| Key 3 | The E minor **key** had not been a good choice | guitar | Y |
| Knight 1 | The armour was shiny after the **knight** polished it | clean | Y |
| Knight 2 | This fairytale was about a princess and a **knight** | extra | N |
| Knight 3 | The horse galloped off under the instruction of the **knight** | stallion | Y |
| Lace 1 | The teacher stopped Lily to tie up her **lace** | student | Y |
| Lace 2 | John had forgotten to do the **lace** up on his shoe | language | N |
| Lace 3 | He tripped on the **lace** of one of his new shoes | fell | Y |
| Landing 1 | There was very little space on the first floor **landing** | snail | N |
| Landing 2 | She stood by the stairs on the **landing**, looking down | float | N |
| Landing 3 | Shelves cluttered the limited space at the top of the stairs, on the **landing** | storage | Y |
| Letter 1 | The little girl sounded the word out one **letter** at a time | stamp | N |
| Letter 2 | The English alphabet is a 26-**letter** Latin alphabet | language | Y |
| Letter 3 | When typing his name, George had missed a **letter** out | grab | N |
| Mark 1 | The lecturer hadn’t had time to give the essay a **mark** | chew | N |
| Mark 2 | He was extremely disappointed with his average **mark** | essay | Y |
| Mark 3 | Through omitting an important argument, Sophie had lost another **mark** | milk | N |
| Match 1 | The derby was certainly going to be an exciting **match** | rivals | Y |
| Match 2 | The team won the **match** against their rivals | sport | Y |
| Match 3 | He trained harder for this **match** than any other in his career | fax | N |
| Mould 1 | He wanted to use tools to help him **mould** the statue | follow | N |
| Mould 2 | You can easily **mould** a clay-like substance | obtain | N |
| Mould 3 | The artist had to **mould** the substance into the right shape | sculpture | Y |
| Mouse 1 | The Apple iMac boasts a new **mouse** or track-pad device | technology | Y |
| Mouse 2 | The machine comes with a state-of-the-art **mouse** | shelter | N |
| Mouse 3 | The wireless **mouse** is ergonomically designed, so that it can be used with comfort | pin | N |
| Nail 1 | She was upset that she had broken her **nail** | woman | Y |
| Nail 2 | He stubbed his toe and split his toe **nail** | cheer | N |
| Nail 3 | The varnish had chipped off one **nail** | zoom | N |
| Note 1 | The lower **note** suited the singer’s voice better than the higher one | require | N |
| Note 2 | The violinist held the **note** for as long as possible | string | Y |
| Note 3 | Since his voice had broken, he was no longer able to reach the **note** | sing | Y |
| Organ 1 | The young woman wanted to learn how to play the **organ** | musician | Y |
| Organ 2 | The church was filled with the sound of the **organ** | hammer | N |
| Organ 3 | The musician played the **organ** beautifully | church | Y |
| Pair 1 | Since the heel was broken, she bought a new **pair** of shoes | stiletto | Y |
| Pair 2 | The couple always came to events as a **pair** | eye | N |
| Pair 3 | Her wellies were still wet so she had to borrow a **pair** from her sister | soaked | Y |
| Palm 1 | The start of the private beach was indicated with a **palm** | bite | N |
| Palm 2 | The bathers sought shade under the **palm** | heat | Y |
| Palm 3 | The only respite from the midday heat was under the **palm** | beach | Y |
| Panel 1 | There was a temporary wooden **panel** separating the two rooms | wall | Y |
| Panel 2 | The car had a dent on the side **panel** | hop | N |
| Panel 3 | The insulator was placed between the wall and the **panel** | bread | N |
| Park 1 | With his new car, he struggled to **park** | ordinary | N |
| Park 2 | The learner driver was taught how to parallel **park** | lesson | Y |
| Park 3 | There wasn’t a space in which to **park** her car | driving | Y |
| Pen 1 | The pig **pen** was muddier than ever | animals | Y |
| Pen 2 | The farm kept too many animals in each **pen** | pink | N |
| Pen 3 | The farmer put the animals in the **pen** | riddle | N |
| Pipe 1 | The grandfather picked up his **pipe** to smoke | old | Y |
| Pipe 2 | There was already a strong smell of tobacco in the **pipe** | smoke | Y |
| Pipe 3 | The men smoked a **pipe** while they waited | elbow | N |
| Plug 1 | The plumber had forgotten to put the **plug** in | chalk | N |
| Plug 2 | The cleaner poured bleach down the **plug** | hygienic | Y |
| Plug 3 | In the bath, a spider was near the **plug** hole | bathroom | Y |
| Punch 1 | The guests made the most of the free **punch** | roast | N |
| Punch 2 | The host offered everyone some homemade **punch** | alcohol | Y |
| Punch 3 | The fruit **punch** had been spilt over the table | accident | Y |
| Pupil 1 | Lizzie was the best **pupil** in the class | exemplary | Y |
| Pupil 2 | A former **pupil** returned to the school to give a careers talk | robust | N |
| Pupil 3 | The teacher helped the new **pupil** | bell | N |
| Racket 1 | Adam turned the TV up because of the **racket** | ink | N |
| Racket 2 | He couldn’t hear over the **racket** | listen | Y |
| Racket 3 | At lunch time the school children made such a **racket** | chat | Y |
| Record 1 | Today was the hottest day on **record** | timber | N |
| Record 2 | It was by far the busiest film event on **record** | hose | N |
| Record 3 | The athlete broke the world **record** for the long jump | winner | Y |
| Ring 1 | She picked up the phone to give her daughter a **ring** | fry | N |
| Ring 2 | He checked his phone to find out if his friend had given him a **ring** | hotel | N |
| Ring 3 | To check that she was ok, the office gave her a **ring** | employee | Y |
| See 1 | He sat up straight but still couldn’t **see** | warm | N |
| See 2 | Lily went to **see** what was going on | investigate | Y |
| See 3 | It was really easy to **see** what had gone wrong | error | Y |
| Sign 1 | After giving her name and the date, she had to **sign** the contract | legal | Y |
| Sign 2 | When the parcel was delivered, someone had to **sign** for it | joke | N |
| Sign 3 | At the end of the document, he was asked to **sign** | throne | N |
| Sink 1 | There was a soap pump on the side of the **sink** | wriggle | N |
| Sink 2 | While the shower was being fixed, he washed in the **sink** | still | N |
| Sink 3 | Water had dripped out the bottom of the **sink** and onto the floor | leak | Y |
| Skip 1 | The home owners were advised to hire a **skip** | rubbish | Y |
| Skip 2 | The builder threw the old bricks into the **skip** | internet | N |
| Skip 3 | The cat had slept on an old mattress that had been dumped in a **skip** | stray | Y |
| Son 1 | The mother made a packed lunch for her **son** | income | N |
| Son 2 | The father and **son** looked alike | super | N |
| Son 3 | They looked at a university for their **son** | degree | Y |
| Spade 1 | The gambler knew that his opponent wanted a **spade** | pint | N |
| Spade 2 | The dealer hadn’t dealt a **spade** yet | game | Y |
| Spade 3 | The gambler would win the game if he got one more **spade** | harm | N |
| Speaker 1 | Dan connected his iPhone to the **speaker** | music | Y |
| Speaker 2 | The sound from the new **speaker** was too loud | heart | N |
| Speaker 3 | The music company released their own brand of **speaker** | stew | N |
| Spring 1 | To make it jump, the toy had a **spring** inside it | marry | N |
| Spring 2 | The toy was broken because of a rusty **spring** | oath | N |
| Spring 3 | The suspension was improved with a different **spring** | car | Y |
| Staff 1 | When walking up-hill, the hiker used a **staff** to help | mountain | Y |
| Staff 2 | A wooden **staff** was propped up in the corner of the entrance hall | mad | N |
| Staff 3 | The shepherd had to use a **staff** | sheep | Y |
| Step 1 | She daren’t take one more **step** in the area | sugar | N |
| Step 2 | He couldn’t take another **step** forward without his foot hurting | stir | N |
| Step 3 | The mother asked him to move a **step** closer | request | Y |
| Stitch 1 | The athlete was in a lot of pain because of the **stitch** | exercise | Y |
| Stitch 2 | The dancer stretched to ease the pain of the **stitch** | hurt | Y |
| Stitch 3 | The runner had to push through the **stitch** to try and win the race | sand | N |
| Straw 1 | The foal was born on the **straw** in the barn | horse | Y |
| Straw 2 | The stable floor was covered in good quality **straw** | farm | Y |
| Straw 3 | The children were asked to feed the animals with **straw** | tender | N |
| Strike 1 | A policy amendment led to a **strike** across the profession | union | Y |
| Strike 2 | All the workers went on **strike** | consist | N |
| Strike 3 | The union helped to prepare for the **strike** | crib | N |
| Temple 1 | One of the pressure points of the body is the **temple** | comb | N |
| Temple 2 | The doctor had a headache around her **temple** | stress | Y |
| Temple 3 | To meditate, the man held his fingers on his **temple** | angle | N |
| Toast 1 | The host was asked to make a **toast** | speech | Y |
| Toast 2 | The guests held up their drinks to **toast** the couple | purpose | N |
| Toast 3 | They made a **toast** to her for her hard work | celebration | Y |
| Trailer 1 | The new film **trailer** was released yesterday | snore | N |
| Trailer 2 | Scenes from the film are shown in the **trailer** | movie | Y |
| Trailer 3 | The soundtrack used in the **trailer** has already reached number one | bench | N |
| Trunk 1 | Pat lifted the lid of the **trunk** to check what was inside | look | Y |
| Trunk 2 | The papers were hidden inside the **trunk** | secret | Y |
| Trunk 3 | The children’s dressing up clothes were kept in a **trunk** | gold | N |
| Watch 1 | The boys tried to **watch** from their treehouse | children | Y |
| Watch 2 | Rebecca wanted to **watch** the puppy all day | peck | N |
| Watch 3 | The new employees had to **watch** an introductory video | training | Y |
| Wave 1 | He couldn’t hear what she said but he saw her **wave** | greeting | Y |
| Wave 2 | Alex stood by the window to **wave** goodbye | blink | N |
| Wave 3 | She tried to get the celebrity’s attention and wanted her to **wave** back | partner | N |
